# Supplementary material for: A natural fusion of flavodiiron, rubredoxin, and rubredoxin oxidoreductase domains is a self-sufficient water-forming oxidase of Trichomonas vaginalis
Source: J Biol Chem. 2022 Jun 30;298(8):102210. doi: 10.1016/j.jbc.2022.102210 (PMC9364112; doi:10.1016/j.jbc.2022.102210)
Supplement: Supporting Information [file mmc1.docx]

**References**

63. Hayashi, T., Caranto, J. D., Wampler, D. A., Kurtz, D. M., and Moenne-Loccoz, P. (2010) Insights into the nitric oxide reductase mechanism of flavodiiron proteins from a flavin-free enzyme. *Biochemistry* **49**, 7040-7049

64. Silaghi-Dumitrescu, R., Ng, K. Y., Viswanathan, R., and Kurtz, D. M., Jr. (2005) A flavo-diiron protein from Desulfovibrio vulgaris with oxidase and nitric oxide reductase activities. Evidence for an in vivo nitric oxide scavenging function. *Biochemistry* **44**, 3572-3579

65. Hillmann, F., Riebe, O., Fischer, R. J., Mot, A., Caranto, J. D., Kurtz, D. M., Jr., and Bahl, H. (2009) Reductive dioxygen scavenging by flavo-diiron proteins of Clostridium acetobutylicum. *FEBS Lett* **583**, 241-245

66. Saeki, K., Jain, M. K., Shen, G. J., Prince, R. C., and Zeikus, J. G. (1989) Purification and properties of ferredoxin and rubredoxin from Butyribacterium methylotrophicum. *J Bacteriol* **171**, 4736-4741

67. Yoon, K. S., Hille, R., Hemann, C., and Tabita, F. R. (1999) Rubredoxin from the green sulfur bacterium Chlorobium tepidum functions as an electron acceptor for pyruvate ferredoxin oxidoreductase. *J Biol Chem* **274**, 29772-29778

68. Swartz, P. D., Beck, B. W., and Ichiye, T. (1996) Structural origins of redox potentials in Fe-S proteins: electrostatic potentials of crystal structures. *Biophys J* **71**, 2958-2969

69. Vicente, J. B., and Teixeira, M. (2005) Redox and spectroscopic properties of the Escherichia coli nitric oxide-detoxifying system involving flavorubredoxin and its NADH-oxidizing redox partner. *J Biol Chem* **280**, 34599-34608

70. Pierik, A. J., Wolbert, R. B., Portier, G. L., Verhagen, M. F., and Hagen, W. R. (1993) Nigerythrin and rubrerythrin from Desulfovibrio vulgaris each contain two mononuclear iron centers and two dinuclear iron clusters. *European journal of biochemistry / FEBS* **212**, 237-245

71. Grunden, A. M., Jenney, F. E., Jr., Ma, K., Ji, M., Weinberg, M. V., and Adams, M. W. (2005) In vitro reconstitution of an NADPH-dependent superoxide reduction pathway from Pyrococcus furiosus. *Appl Environ Microbiol* **71**, 1522-1530

72. Lee, H. J., Basran, J., and Scrutton, N. S. (1998) Electron transfer from flavin to iron in the Pseudomonas oleovorans rubredoxin reductase-rubredoxin electron transfer complex. *Biochemistry* **37**, 15513-15522

73. Gomes, C. M., Vicente, J. B., Wasserfallen, A., and Teixeira, M. (2000) Spectroscopic studies and characterization of a novel electron-transfer chain from Escherichia coli involving a flavorubredoxin and its flavoprotein reductase partner. *Biochemistry* **39**, 16230-16237

**Supporting Figure Legends:**

***Supplementary Figure S1.*** Multiple sequence alignment of the N-terminal sequences of Class F flavodiiron proteins from *T. vaginalis* (*Tv*FDPF1-3) (TVAG_263800, TVAG_049830, TVAG_121610) and "stand-alone" Class A FDPs from *T. vaginalis* (TVAG_036010), *Giardia intestinalis* (XP_001707670), *Entamoeba histolytica* (XP_651627), *Entamoeba dispar* (XP_001738262), *Entamoeba moshkovskii* (CAI11385), *Desulfovibrio gigas* (WP_021760300) and *Desulfovibrio vulgaris* (WP_010940443). Conserved residues which are important for the binding of both irons of the diiron center are highlighted in red: Fe 1 (His82-X-Glu84-X-Asp86-His87); Fe 2 (His148-X_18_-Asp166-X_64_-His230).

***Supplementary Figure S2.*** Multiple sequence alignment of the rubredoxin-like domain of *Tv*FDPF1-3, “stand-alone” rubredoxins from *Thermotoga maritima* (AAD35743), *Clostridium acetobutylicum* (Q9AL94) and *Pyrococcus abyssi* (WP_010868015) and the C-terminal domains of rubrerythrin from *Desulfovibrio vulgaris* (WP_010940353) and *Clostridium perfringens* (WP_164819022). Conserved cysteine residues which form the Fe(SCys)_4_ center are highlighted. In both *Tv*FDPF1-3 and rubrerythrin the conserved sequence is CXXC-X_12_-CXXC, which is different from the one found in a classical rubredoxin (CXXC-X_29_-CXXC).

***Supplementary Figure S3.*** Multiple sequence alignment of the C-terminal NADH:rubredoxin oxidoreductase-like domains of *Tv*FDPF1-3, “stand-alone” NADH:rubredoxin oxidoreductase from *Thermotoga maritima* (WP_004080954), NADH:ferredoxin oxidoreductase from *Novosphingobium aromaticivorans* (ABD24664), H_2_O-forming oxidases from *Lactobacillus brevis* (BAN07126) and *Thermus thermophilus* (WP_011173859) and CoA-disulfide reductase from *Bacillus anthracis* (WP_000087591). Both the dinucleotide-binding motif and NAD(P)H substrate binding loop are highlighted.

***Supplementary Figure S4.*** ***Initial purification of TvFDPF1-3 and their spectroscopic and kinetic analysis.* (a)** Purified *Tv*FDPF1 (lane 1), *Tv*FDPF2 (lane 2) and *Tv*FDPF3 (lane 3) (7 μg per lane). **(b-c)** UV-visible spectra of purified *Tv*FDPF1 and 3. Proteins were in buffer E at 40 μM (calculated based on the molecular weight of a monomer) as purified and after addition of 3 mM of sodium dithionite under aerobic conditions (bleached absorbance). The UV-visible spectrum of *Tv*FDPF2 suffers from extensive protein aggregation (shown in Supplementary Figure S5). **(d-f)** Michaelis-Menten analysis of the oxidase activity of *Tv*FDPF1-3 as described under “Experimental procedures” with NADH (filled circles) and NADPH (open circles). All kinetic parameters for *Tv*FDPF1-3 are summarized in Table 2.

***Supplementary Figure S5.*** ***UV-visible spectra of purified TvFDPF2.*** Protein was in buffer E at 40 μM (calculated based on the molecular weight of a monomer) as purified and after addition of 3 mM of sodium dithionite under aerobic conditions. Aggregation of the protein can be seen by severe upward shift in the spectrum.

***Supplementary Figure S6.*** Activity with NO was quantified as described under “Experimental procedures”. We estimated that the NOase activity of *Tv*FDPF3 was <2% of the NADH to O_2_ specific activity.

***Supplementary Figure S7. Kinetic characterization of the reaction with oxygen.*** Simultaneous measurements of oxygen (black traces) and NADH (blue traces) consumption by *Tv*FDPF1 (**a**) and *Tv*FDPF3 (**b**) that were performed as described under “Experimental procedures”. Additions of NADH and enzymes are indicated with arrows.

***Supplementary Figure S8.*** BN-PAGE of holo*Tv*FDPF3 (lane 1), *Lb*NOX (lane 2) and *Gi*NOX (lane 3). Based on the analytical gel-filtration the apparent molecular weight of *Lb*NOX is 195 ± 3 kDa and of *Gi*NOX is 241 ± 1 kDa. Note that both *Lb*NOX and *Gi*NOX migrated on BN-PAGE accordingly to their expected molecular weights as determined by gel-filtration. The apparent molecular weight of holo*Tv*FDPF3 based on BN-PAGE is 322 ± 24 kDa based on (n = 3) independent experiments ± s.d.

***Supplementary Figure S9.*** Original EPR spectra for one of the titrations of holo*Tv*FDPF3 that depict the *g*=4.3 feature used for determination of the redox potential of the rubredoxin center. Redox potentials at which spectra were recorded are indicated.

***Supplementary Figure S10.*** Spectra recorded during the course of the UV-Vis titration of holo*Tv*FDPF3 (**a**) and deFMN-*Tv*FDPF3 (**b**). Redox potentials at which spectra were recorded are indicated.

***Supplementary Figure S11.* (a)** Visible spectrum of the neutral semiquinone of FAD upon redox titration of deFMN-*Tv*FDPF3. Protein sample was poised -232 mV. The contribution of oxidized and reduced FAD (30% each) was subtracted to reveal the features of the semiquinone only. **(b)** Normalized semiquinone intensities as a function of separation of the FADH_2_/FADH ^•^ and FADH^•^/FAD redox potentials. At values below 60 mV the shape no longer reveals the separation of potentials. **(c)** Absolute intensity of the semiquinone as a function of separation of the FADH_2_/FADH ^•^ and FADH^•^/FAD redox potentials. The arrow indicates semiquinone content for deFMN-*Tv*FDPF3 as estimated from the absorbance at 600 nm.

***Supplementary Figure S12.*** ***Simplified cryo-EM data processing workflow.***

***Supplementary Figure S13.*** ***Cryo-EM map validation (C1 map).* (a)** Local resolution profile for the masked and sharpened C1 (no symmetry applied) holo*Tv*FDPF3 map. **(b)** Fourier shell correlation for the unmasked, masked, and phase-randomized masked C1 maps. The final corrected FSC is indicated in black. **(c)** Angular distribution of particle views in the final refined holo*Tv*FDPF3 C1 map.

***Supplementary Figure S14. Homology model docking in C1 map.* (a)** C1-symmetric (no symmetry applied) electron density map for the holo*Tv*FDPF3 particles. **(b)** Homology models for the large and small subdomains of the holo*Tv*FDPF3 FDP-like domain docked into the C1-symmetric density map. The placement of the models indicates apparent C2 symmetry.

***Supplementary Figure S15.* *Cryo-EM Map Validation (C2 Map).* (a)** Local resolution profile for the masked and sharpened C2-symmetric holo*Tv*FDPF3 map. **(b)** Fourier shell correlation for the unmasked, masked, and phase-randomized masked C2 maps. The final corrected FSC is indicated in black. **(c)** Angular distribution of particle views in the final refined holo*Tv*FDPF3 C2 map.

***Supplementary Figure S16. Ligand occupancy in electron density.*** Close-up view of the **(a)** FMN and **(b)** diiron ligands in the C2-symmetric electron density map.

***Supplementary Figure S17.* *Inter-subunit contacts between holoTvFDPF3 protomers.*** Homology model of one subunit of the holo*Tv*FDPF3 FDP-like domain colored to identify interfacial residues (defined as residues in one subunit within 3.5 Å of any residue in the neighboring subunit). Residues colored blue form inter-protomer contacts in both the holo*Tv*FDPF3 model and the published structure of *D. gigas* ROO. Residues colored red form inter-protomer contacts unique to holo*Tv*FDPF3. Residues colored in purple form inter-protomer contacts in *D. gigas* ROO, but not in the model of holo*Tv*FDPF3. Residues are listed as numbered in holo*Tv*FDPF3.

**SUPPORTING TABLES**

**Supplementary Table S1.**

Flavodiiron proteins found in human microaerophilic parasite *T. vaginalis.*

| **Accession ID** | **Current annotation in EuPathDB database** | **FDP Class** | **Name used**  **in this study** | **Mol. mass**  **(monomer)*,**  **kDa** | **pI*** |
| --- | --- | --- | --- | --- | --- |
| TVAG_263800 | disulfide oxidoreductase, putative | Class F | *Tv*FDPF1 | 95.5 | 5.25 |
| TVAG_049830 | disulfide oxidoreductase, putative | Class F | *Tv*FDPF2 | 94.9 | 5.75 |
| TVAG_121610 | apoptosis inducing factor, putative | Class F | *Tv*FDPF3 | 96.0 | 5.63 |
| TVAG_036010 | A-type flavoprotein | Class A | *Tv*FDPA | 46.3 | 7.95 |

*****both values were computed using Expasy Swiss Bioinformatics Resource Portal. Class F FDPs from *T. vaginalis* (*Tv*FDPF1-3) are currently misannotated in EuPathDB and GeneBank databases. Molecular masses, pIs, and the V_max_ of the most active holo*Tv*FDPF3 (see Table 2) from this study are strikingly similar to biochemical properties of the native *T. vaginalis* H_2_O-forming NADH oxidase activity purified by Linstead and Bradley in 1988 (32). M.M.- molecular mass; pI – isoelectric point.

**Supplementary Table S2.**

Iron content of *Tv*FDPF1-3.

| **Protein** | **Fe per protein monomer** | **% loading**  **(100% = 3 Fe per monomer)** |
| --- | --- | --- |
| ***Tv*FDPF1** | 0.82 ± 0.17 | 27 ± 6 |
| ***Tv*FDPF2** | 0.41 ± 0.02 | 14 ± 1 |
| ***Tv*FDPF3** | 0.58 ± 0.10 | 19 ± 3 |
| **holo*Tv*FDPF3** | 1.70 ± 0.12 | 57 ± 4 |
|  |  |  |

Iron content was quantified as described under “Experimental procedures”. Stoichiometry was calculated per protein monomers. Data represents (n=4-6) independent experiments ± s.d.

**Supplementary Table S3.**

H_2_O_2_ production by *Tv*FDPF1-3.

| **Protein** | **H_2_O_2_ production,**  **(%)** |
| --- | --- |
| ***Tv*FDPF1** | 86 ± 5 |
| ***Tv*FDPF2** | 25 ± 2 |
| ***Tv*FDPF3** | 9.6 ± 3.7 |
| ***holoTv*FDPF3** | 5.0 ± 2.7 |
| ***Lb*NOX** | 0.6 ± 0.1 |

H_2_O_2_-formation was monitored in AmplexRed-based assay at 37^o^C as described under “Experimental procedures”. Estimated H_2_O_2_ formation is shown as % of the reaction of NADH to NAD^+^ conversion based on (n = 5) independent experiments ± s.d.

**Supplementary Table S4.**

Metal content of holo*Tv*FDPF3 determined by ICP-MS.

| **Metal** | **Metal per protein monomers** |
| --- | --- |
| **Fe** | 2.24 ± 0.03 |
| **Cr** | 0.001 ± 0.00004 |
| **Mn** | 0.0091 ± 0.00015 |
| **Cu** | -0.00104 ± 0.00009 |
| **Co** | 0.00081 ± 0.00003 |
| **Zn** | 0.110 ± 0.001 |

Metal content was determined in by Inductively Coupled Plasma Mass Spectrometry (ICP-MS) in holo*Tv*FDPF3 protein sample diluted to 0.5 μM of protein monomers. Values are based on (n=6) technical replicates.

**Supplementary Table S5.**

Cryo-EM data collection and processing statistics.

| **Accessions** | | |
| --- | --- | --- |
|  | **C1 Symmetric Map** | **C2 Symmetric Map** |
| **EMDB (EM Maps) ID** | EMD-25790 | EMD-25787 |
| **EMPIAR (EM Images) ID** | EMPIAR-10895 | |
| **Zenodo (Docked Homology Models) Accession** | 10.5281/zenodo.5795907 | |
| **Data Collection and Processing** | | |
| **Microscope** | Titan Krios | |
| **Camera** | K3 | |
| **Magnification** | 105,000 x | |
| **Voltage (kV)** | 300 | |
| **Total Electron Dose (e^-^ / Å^2^)** | 54.5 | |
| **Defocus range (μm)** | -1.3 to -2.5 | |
| **Pixel size (Å)** | 0.825 | |
| **Micrographs collected** | 7,398 | |
| **Final particles** | 101,628 | 53,709 |
| **Symmetry** | C1 | C2 |
| **Resolution (Å, FSC 0.143)** | 6.6 | 6.8 |

**Supplementary Table S6.**

Activities of representative flavodiiron proteins.

| Protein | oxidase activity*, s^-1^ | NOase activity, s^-1^ |
| --- | --- | --- |
| **Class A**  *G. intestinalis* (20) | 37.7 ± 8.3 | 0.2 |
| **Class A**  *E. histolytica* (13) | 400 ± 30 | 1.7 ± 0.4 |
| **Class A**  *T. maritima* (63) | 4 | 0.05 |
| **Class A**  *M. thermoacetica (*64*)* | 50 | 48 |
| **Class A**  *D. vulgaris (*64*)* | 24 | 19 |
| **Class A**  *C. acetobutylicum (*65*)* | 5 | 34 |
| **Class F**  *C. difficile* (39) | 16.0 ± 1.3 | 0.20 ± 0.01 |
| **Class F**  *T. vaginalis holoTv*FDP3 (this study) | 466 ± 42 | N.D. |

*Measured as O_2_ or NADH consumption. In most studies *k*_cat_ are reported for an arbitrary substrate concentration and not necessary under V_max_ conditions. In all assays for recombinant Class A FDPs non-physiological Rb/NROR protein partners were used (NADH:flavorubredoxin oxidoreductase and truncated rubredoxin domain of *E. coli* flavorubredoxin). N.D.- not detected.

| **Protein** | **E1-FAD** | **E2-FAD** | **Average** | **E1-FMN** |  | **E2-FMN** |  | **Average** | **Rb** | **E1-FeFe** |  | **E2-FeFe** |  | **Average** | **Ref.** |
| --- | --- | --- | --- | --- | --- | --- | --- | --- | --- | --- | --- | --- | --- | --- | --- |
|  | **(FADox-FADsq)** | **(FAD**  **sq -FADred)** | **FAD** | **(FMNox-FMNsq)** | **error** | **(FMNsq-FMNred)** | **error** | **FMN** | **Fe^3+^ to Fe^2+^** | **2Fe^3+^ to Fe^3+^/^2+^** | **error** | **Fe^3+^/^2+^ to 2Fe^2+^** | **error** | **FeFe** |  |
| Rubredoxin *C. pasteurianum* |  |  |  |  |  |  |  |  | -57 |  |  |  |  |  | (25) |
| Rubredoxin *B. methylotrophicum* |  |  |  |  |  |  |  |  | -40 |  |  |  |  |  | (66) |
| Rubredoxin *C. tepidum* |  |  |  |  |  |  |  |  | -87 |  |  |  |  |  | (67) |
| Rubredoxin *H. mobilis* |  |  |  |  |  |  |  |  | -46 |  |  |  |  |  | (23) |
| Rubredoxin *P. furiosus* |  |  |  |  |  |  |  |  | 0 |  |  |  |  |  | (21) |
| Rubredoxin *D. vulgaris* |  |  |  |  |  |  |  |  | 0 |  |  |  |  |  | (24) |
| Rubredoxin *D. gigas* |  |  |  |  |  |  |  |  | 6 |  |  |  |  |  | (68) |
| Rubredoxin domain of FlRd  (Class B FDP) *E. coli* |  |  |  |  |  |  |  |  | -123 |  |  |  |  |  | (69) |
| Rubredoxin Domain of FlRd of  *E. coli* with NROR (FlRd-Red) | -250 | -220 | -235 |  | 15 |  | 15 |  | -65 |  |  |  |  |  | (69) |
| Rubrerythrin *D. vulgaris* |  |  |  |  |  |  |  |  | 230 |  |  |  |  |  | (24) |
| Rubrerythrin *D. vulgaris* |  |  |  |  |  |  |  |  | 281 | 339 |  | 246 |  | 292.5 | (70) |
| Nigrerythrin *D. vulgaris* |  |  |  |  |  |  |  |  | 213 | 300 |  | 209 |  | 254.5 | (70) |
| NROR *P. furiosus* | -173 | -173 | -173 |  |  |  |  |  |  |  |  |  |  |  | (71) |
| NROR of *E.* *coli* (FlRd-Red) dithionite as reductant | -255 | -285 | -270 |  | 15 |  | 15 |  |  |  |  |  |  |  | (69) |
| NROR of *E.* *coli* (FlRd-Red) NADH as reductant | -220 | -260 | -240 |  | 15 |  | 15 |  |  |  |  |  |  |  | (69) |
| NROR *P.* *oleovorans* | -247 | -247 | -247 |  |  |  |  |  |  |  |  |  |  |  | (72) |
| FlRd (Class B FDP) *E. coli* |  |  |  | -140 | 15 | -180 | 15 |  | -140 |  |  |  |  |  | (73) |
| FlRd (Class B FDP) *E. coli* |  |  |  | -40 | 15 | -130 | 15 | -85 | -123 | -20 | 20 | -90 | 20 | -55 | (69) |
| FlRd (Class B FDP) *E. coli* with NROR | -250 | -220 | -235 |  |  |  |  |  | -65 |  |  |  |  |  | (69) |
| FDP domain of FlRd (Class B FDP) |  |  |  |  |  |  |  |  |  | 0 | 20 | -50 | 20 | -25 | (69) |
| FprA (Class A FDP) *M. thermoacetica* |  |  |  | -117 | 10 | -220 | 10 | -168.5 |  |  |  |  |  |  | (14) |
| Hrb (Class B FDP) *M. thermoacetica* |  |  |  | -121 | 10 | -121 | 10 | -121 | -30 |  | 10 |  |  |  | (14) |
| Class A FDP *G. intestinalis* |  |  |  | -66 | 15 | -83 | 15 | -74.5 |  | 163 | 20 | 2 | 20 | 82.5 | (35) |
| Class A FDP *T. vaginalis* |  |  |  | 25 |  | 25 |  | 25 |  |  |  |  |  |  | (29) |
| Class A FDP *E. histolytica* |  |  |  | -55 |  | -140 |  | -97.5 |  |  |  |  |  |  | (13) |
| Class F FDP *C. difficile* | -250 | -250 | -250 | -170 |  | -170 |  | -170 | -110 |  |  |  |  |  | (39) |
| Class F FDP *C. difficile NROR domain* | -250 | -250 | -250 |  |  |  |  |  | -130 |  |  |  |  |  | (39) |
| Class F FDP *T. vaginalis* | -229 | -241 | -235 | -146 |  | -146 |  | -146 | -56 | 55 | 30 | -180 | 50 | -62.5 | This study |
| Class F FDP *T. vaginalis* (de-FMN) | -229 | -241 | -235 | - |  | - |  | - | N.D. | N.D. |  | N.D. |  |  |  |
|  |  |  |  |  |  |  |  |  |  |  |  |  |  |  |  |
|  |  | **average** | **-237** |  |  |  |  | **-98** | **-67** |  |  |  |  | **0.8** |  |
|  |  | **Stdev.** | **28** |  |  |  |  | **66** | **50** |  |  |  |  | **72** |  |

**Supplementary Table S7.** Redox potentials of representative rubredoxins, rubrerythrins, NRORs and FDPs. Not for all studies error values were available. Averages and standard deviations calculated for FAD, FMN, rubredoxin and dinuclear iron center refer to the literature data only. Rubrerythrin/nigerythrin values were omitted for the calculation of the average potentials. N.D. - not determined. -, not present.

**SUPPORTING FIGURES**

**
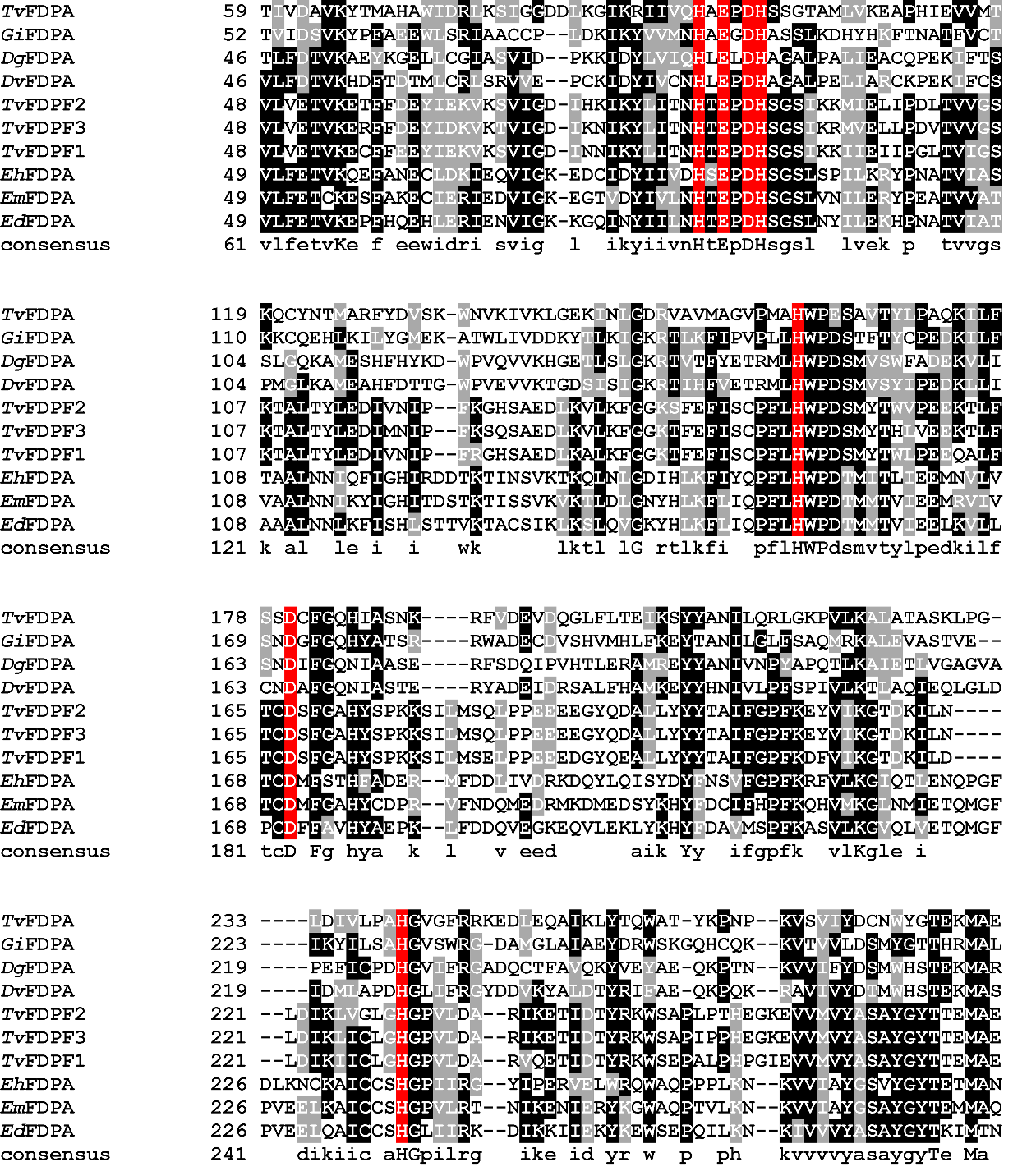
**

***Supplementary Figure S1.*** Multiple sequence alignment of the N-terminal sequences of Class F flavodiiron proteins from *T. vaginalis* (TvFDPF1-3) (TVAG_263800, TVAG_049830, TVAG_121610) and "stand-alone" Class A FDPs from *T. vaginalis* (TVAG_036010) and *Giardia intestinalis* (XP_001707670), *Entamoeba histolytica* (XP_651627), *Entamoeba dispar* (XP_001738262), *Entamoeba moshkovskii* (CAI11385), *Desulfovibrio gigas* (WP_021760300) and *Desulfovibrio vulgaris* (WP_010940443). Conserved residues which are important for the binding of both irons of the diiron center are highlighted in red: Fe 1 (His82-X-Glu84-X-Asp86-His87); Fe 2 (His148-X_18_-Asp166-X_64_-His230).

***
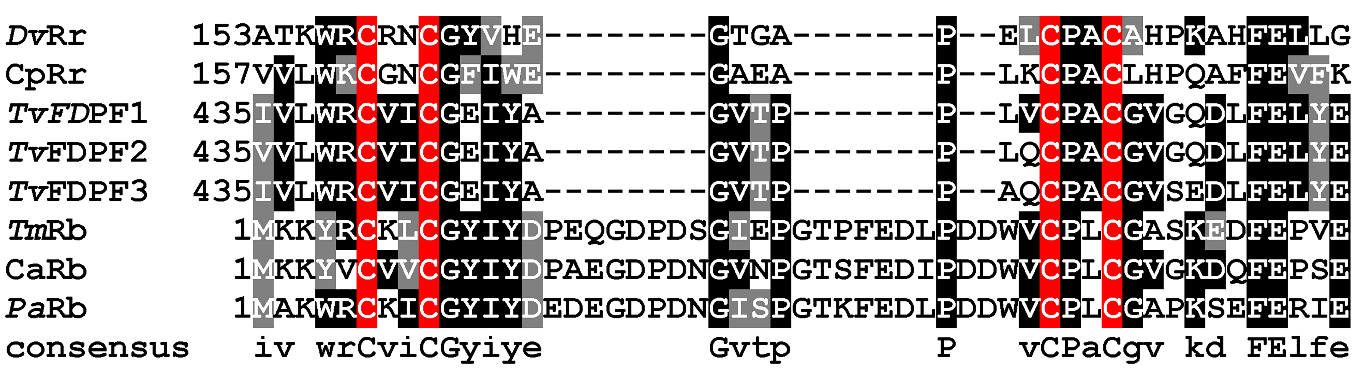
***

***Supplementary Figure S2.*** Multiple sequence alignment of the rubredoxin-like domain of *Tv*FDPF1-3, “stand-alone” rubredoxins from *Thermotoga maritima* (AAD35743), *Clostridium acetobutylicum* (Q9AL94) and *Pyrococcus abyssi* (WP_010868015) and the C-terminal domains of rubrerythrin from *Desulfovibrio vulgaris* (WP_010940353) and *Clostridium perfringens* (WP_164819022). Conserved cysteine residues which form the Fe(SCys)_4_ center are highlighted. In both *Tv*FDPF1-3 and rubrerythrin the conserved sequence is CXXC-X_12_-CXXC, which is different from the one found in a classical rubredoxin CXXC-X_29_-CXXC.

**
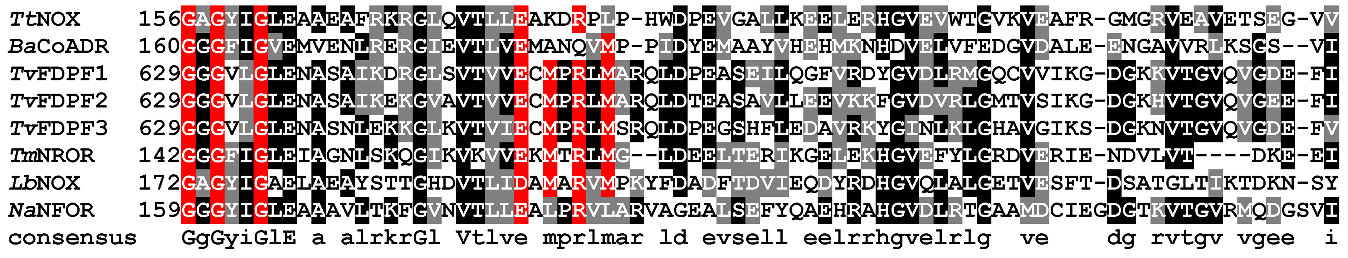
**

***Supplementary Figure S3.*** Multiple sequence alignment of the C-terminal NADH:rubredoxin oxidoreductase-like domains of *Tv*FDPF1-3, “stand-alone” NADH:rubredoxin oxidoreductase from *Thermotoga maritima* (WP_004080954); NADH:ferredoxin oxidoreductase from *Novosphingobium aromaticivorans* (ABD24664); H_2_O-forming oxidases from *Lactobacillus brevis* (BAN07126) and *Thermus thermophilus* (WP_011173859) and CoA-disulfide reductase from *Bacillus anthracis* (WP_000087591). Both the dinucleotide-binding motif and NAD(P)H substrate binding loop are highlighted.

**
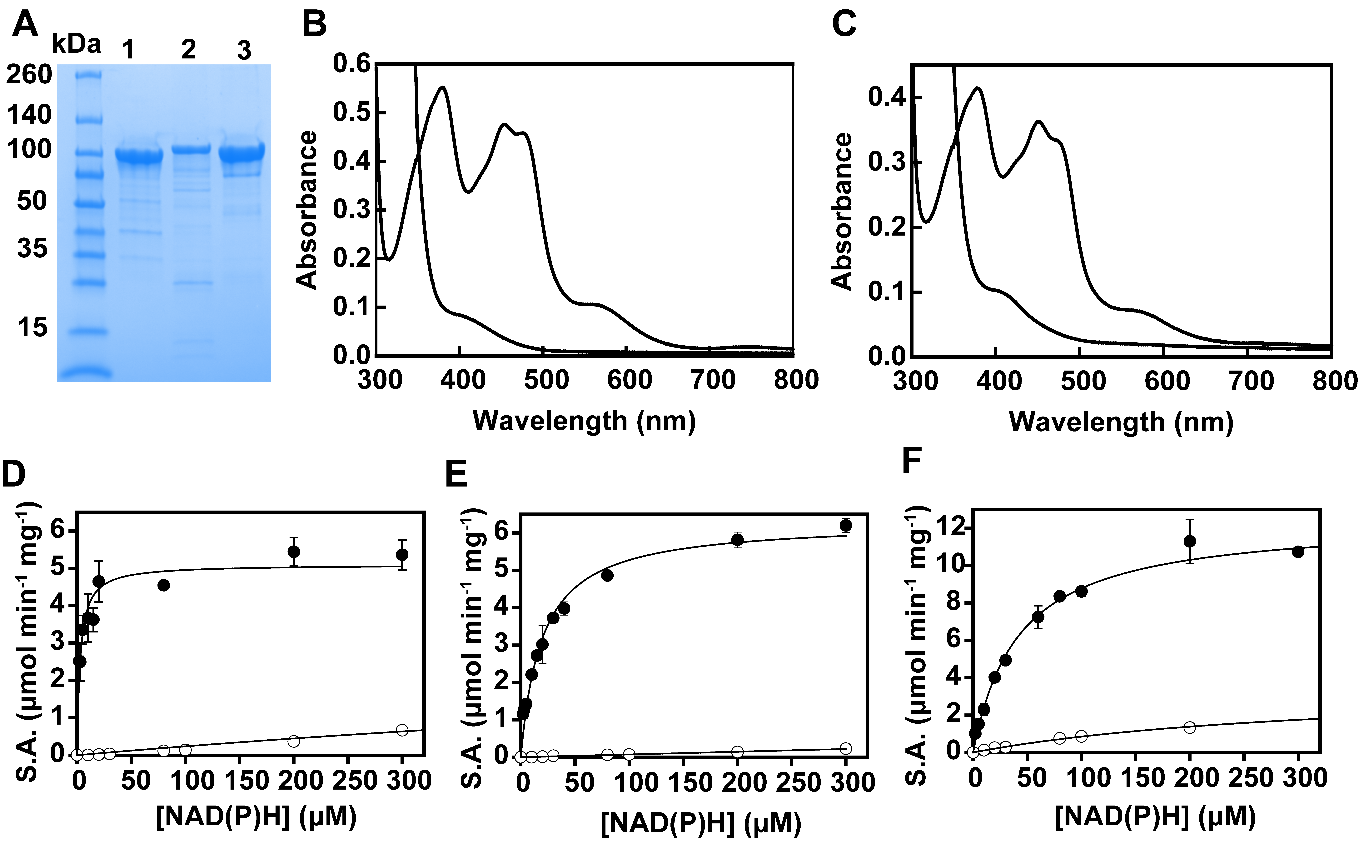
**

***Supplementary Figure S4.*** ***Initial purification of TvFDPF1-3 and their spectroscopic and kinetic analysis.* (a)** Purified *Tv*FDPF1 (lane 1), *Tv*FDPF2 (lane 2) and *Tv*FDPF3 (lane 3) (7 μg per lane). **(b-c)** UV-visible spectra of purified *Tv*FDPF1 and 3. Proteins were in buffer E at 40 μM (calculated based on the molecular weight of a monomer) as purified and after addition of 3 mM of sodium dithionite under aerobic conditions (bleached absorbance). The UV-visible spectrum of *Tv*FDPF2 suffers from extensive protein aggregation (shown in Supplementary Figure S5). **(d-f)** Michaelis-Menten analysis of the oxidase activity of *Tv*FDPF1-3 as described under “Experimental Procedures” with NADH (filled circles) and NADPH (open circles). All kinetic parameters of *Tv*FDPF1-3 are summarized in Table 2.


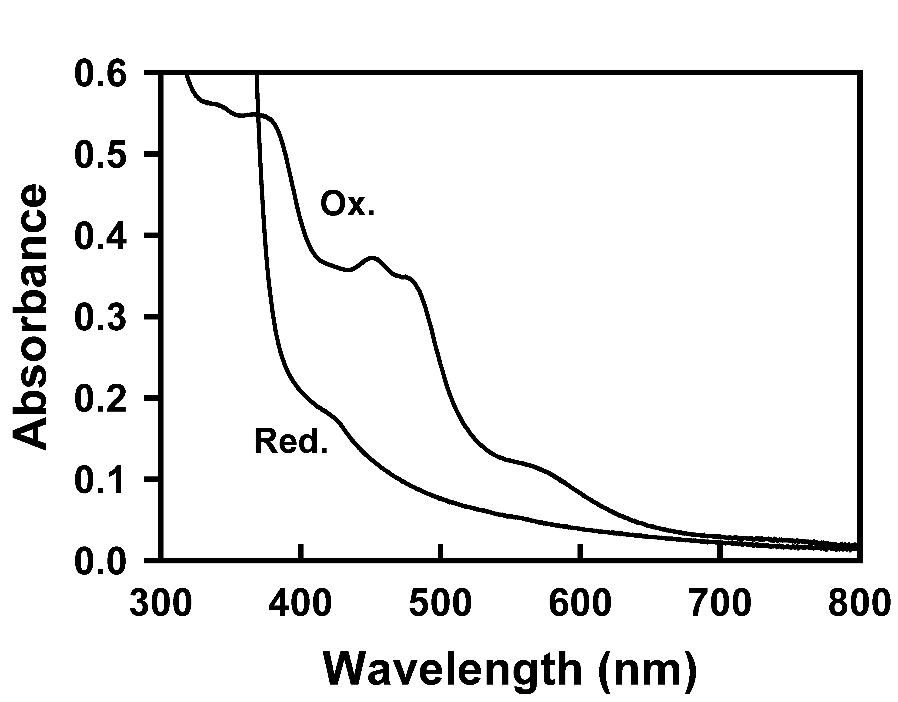


***Supplementary Figure S5.*** ***UV-visible spectra of purified TvFDPF2.*** Protein was in buffer E at 40 μM (calculated based on the molecular weight of a monomer) as purified and after addition of 3 mM of sodium dithionite under aerobic conditions. Aggregation of the protein can be seen by severe upward shift in the spectrum.

**
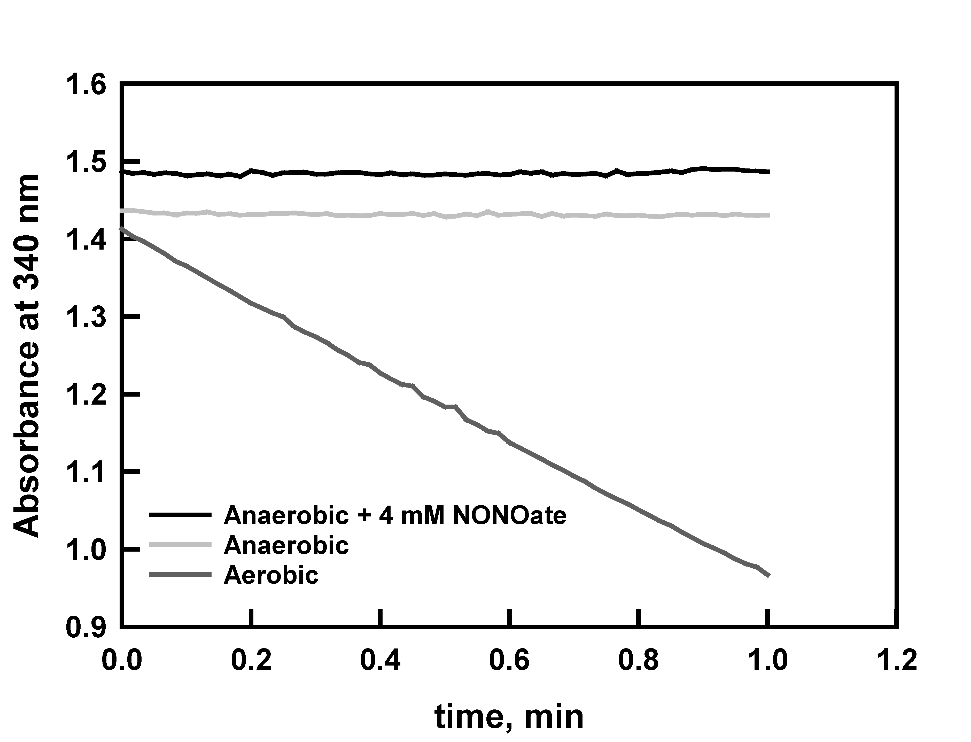
**

***Supplementary Figure S6.*** Activity with NO was quantified as described under “Experimental procedures”. We estimated that the NOase activity of *Tv*FDPF3 was <2% of the NADH to O_2_ specific activity.


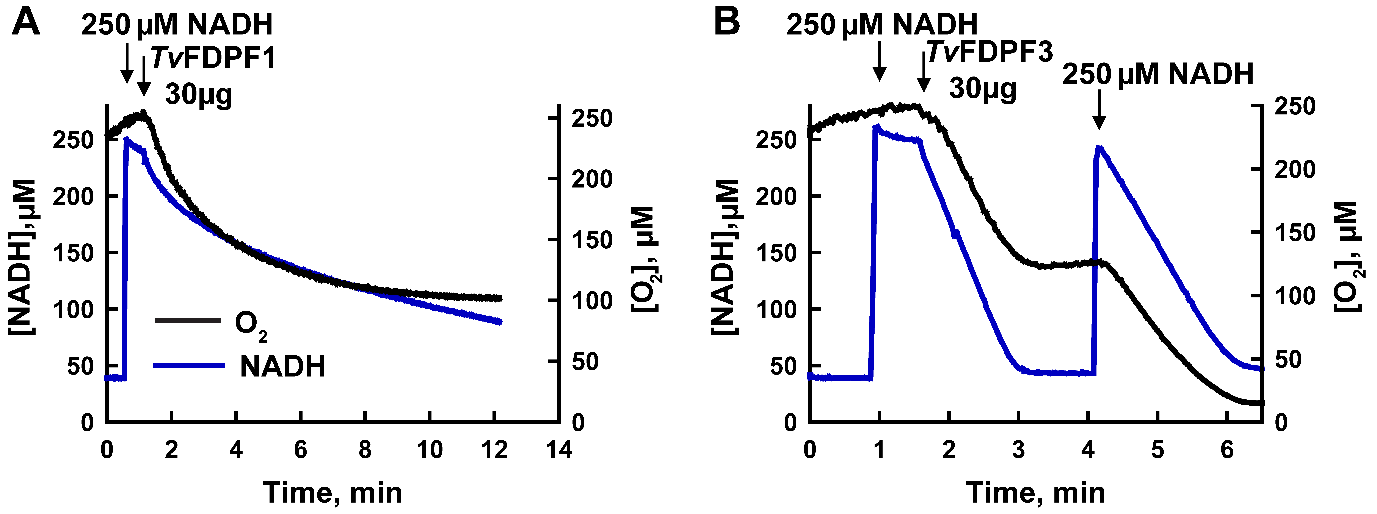


***Supplementary Figure S7. Kinetic characterization of the reaction with oxygen.*** Simultaneous measurements of oxygen (black traces) and NADH (blue traces) consumption by *Tv*FDPF1 (**a**) and *Tv*FDPF3 (**b**) that were performed as described under “Experimental procedures”. Additions of NADH and enzymes are indicated with arrows.

**
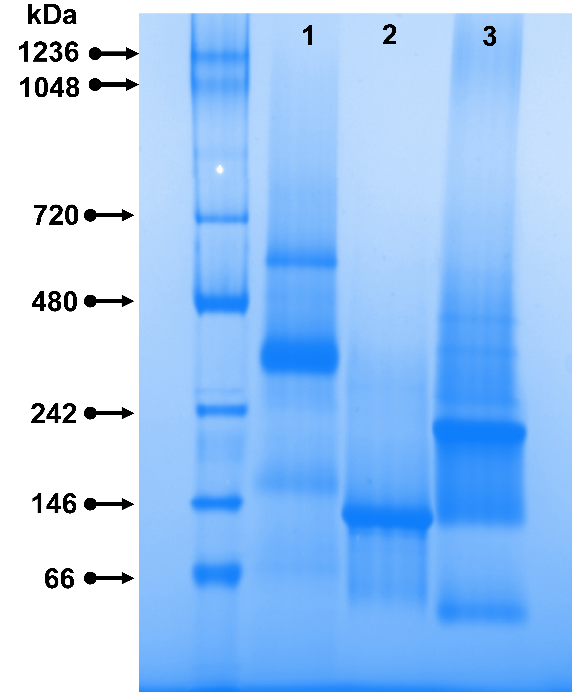
**

***Supplementary Figure S8.*** BN-PAGE of holo*Tv*FDPF3 (lane 1), *Lb*NOX (lane 2) and *Gi*NOX (lane 3). Based on the analytical gel-filtration the apparent molecular weight of *Lb*NOX is 195 ± 3 kDa and of *Gi*NOX is 241 ± 1 kDa. Note both *Lb*NOX and *Gi*NOX migrated on BN-PAGE accordingly to their expected molecular weights as determined by gel-filtration. The apparent molecular weight of holo*Tv*FDPF3 based on BN-PAGE is 322 ± 24 kDa based on (n = 3) independent experiments ± s.d.

**
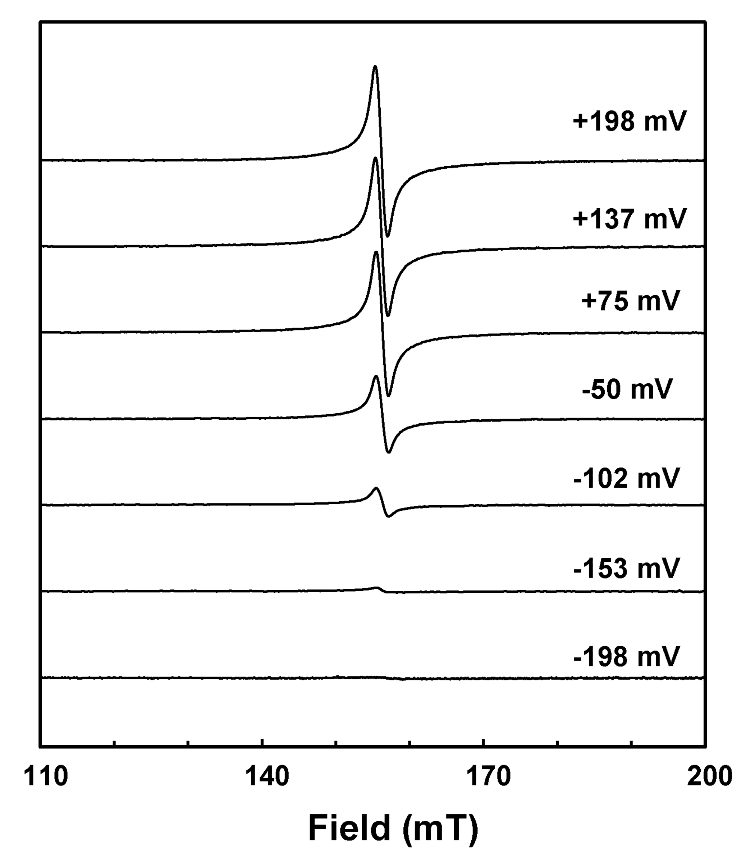
**

***Supplementary Figure S9.*** Original EPR spectra for one of the titrations of holo*Tv*FDPF3, that depict the *g*=4.3 feature used for determination of the redox potential of the rubredoxin center. Redox potentials at which spectra were recorded are indicated.


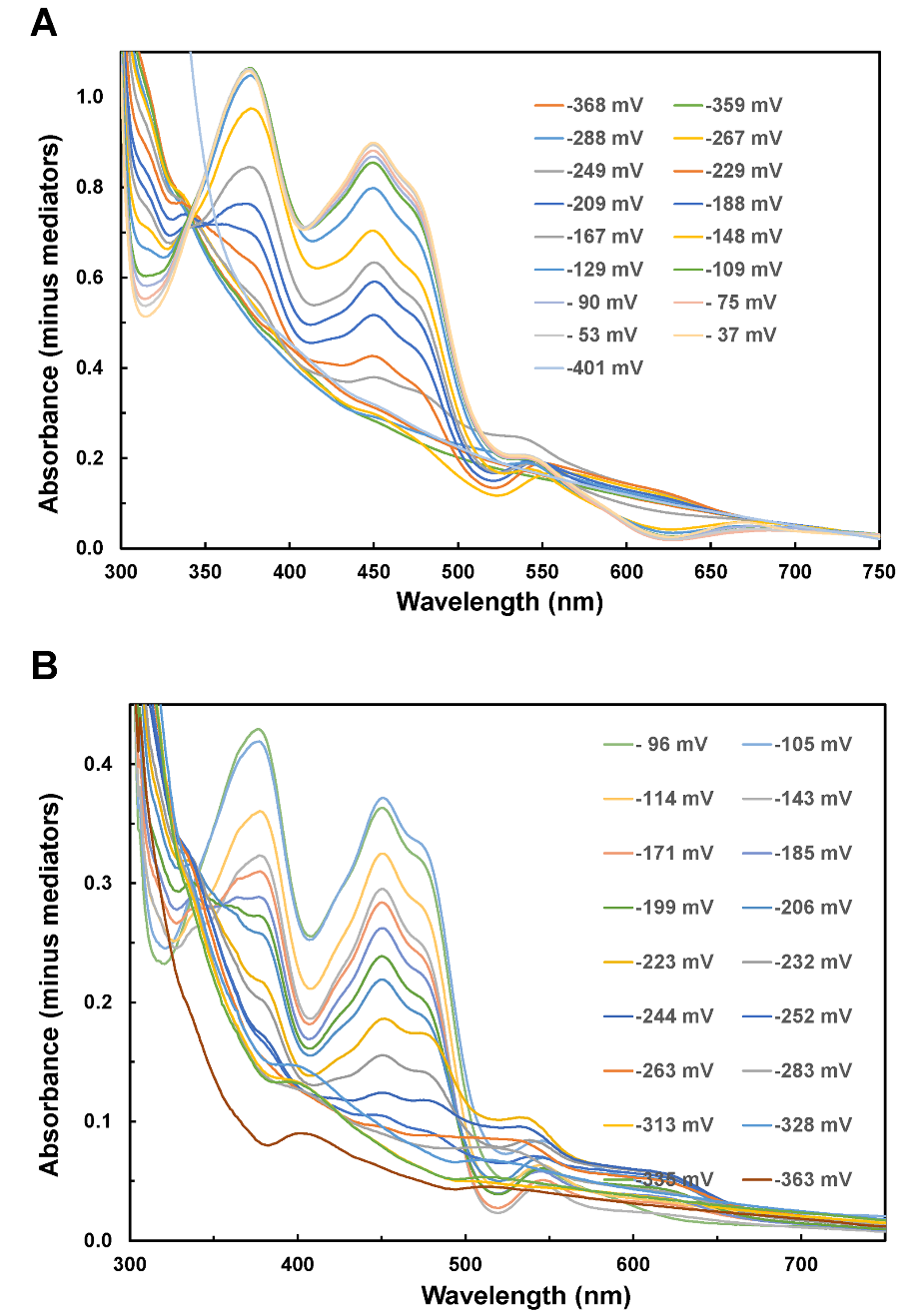


***Supplementary Figure S10.*** Spectra recorded during the course of the UV-Vis titration of holo*Tv*FDPF3 (**A**) and deFMN-*Tv*FDPF3 (**B**). Redox potentials at which spectra were recorded are indicated.

***
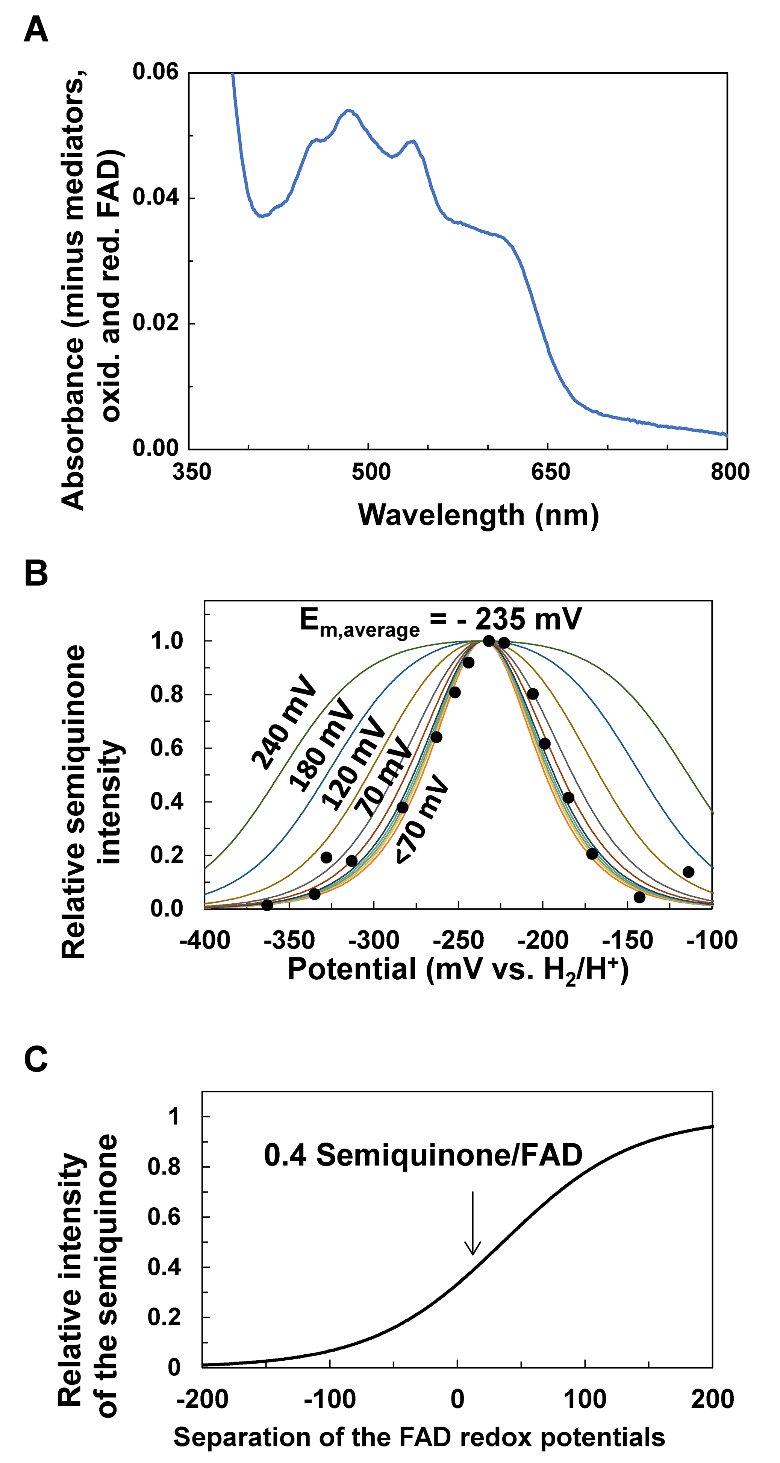
***

***Supplementary Figure S11.* (a)** Visible spectrum of the neutral semiquinone of FAD upon redox titration of deFMN-*Tv*FDPF3. Protein sample was poised -232 mV. The contribution of oxidized and reduced FAD (30% each) was subtracted to reveal the features of the semiquinone only. **(b)** Normalized semiquinone intensities as a function of separation of the FADH_2_/FADH ^•^ and FADH^•^/FAD redox potentials. At values below 60 mV the shape no longer reveals the separation of potentials. **(c)** Absolute intensity of the semiquinone as a function of separation of the FADH_2_/FADH ^•^ and FADH^•^/FAD redox potentials. The arrow indicates semiquinone content for deFMN-*Tv*FDPF3 as estimated from the absorbance at 600 nm.


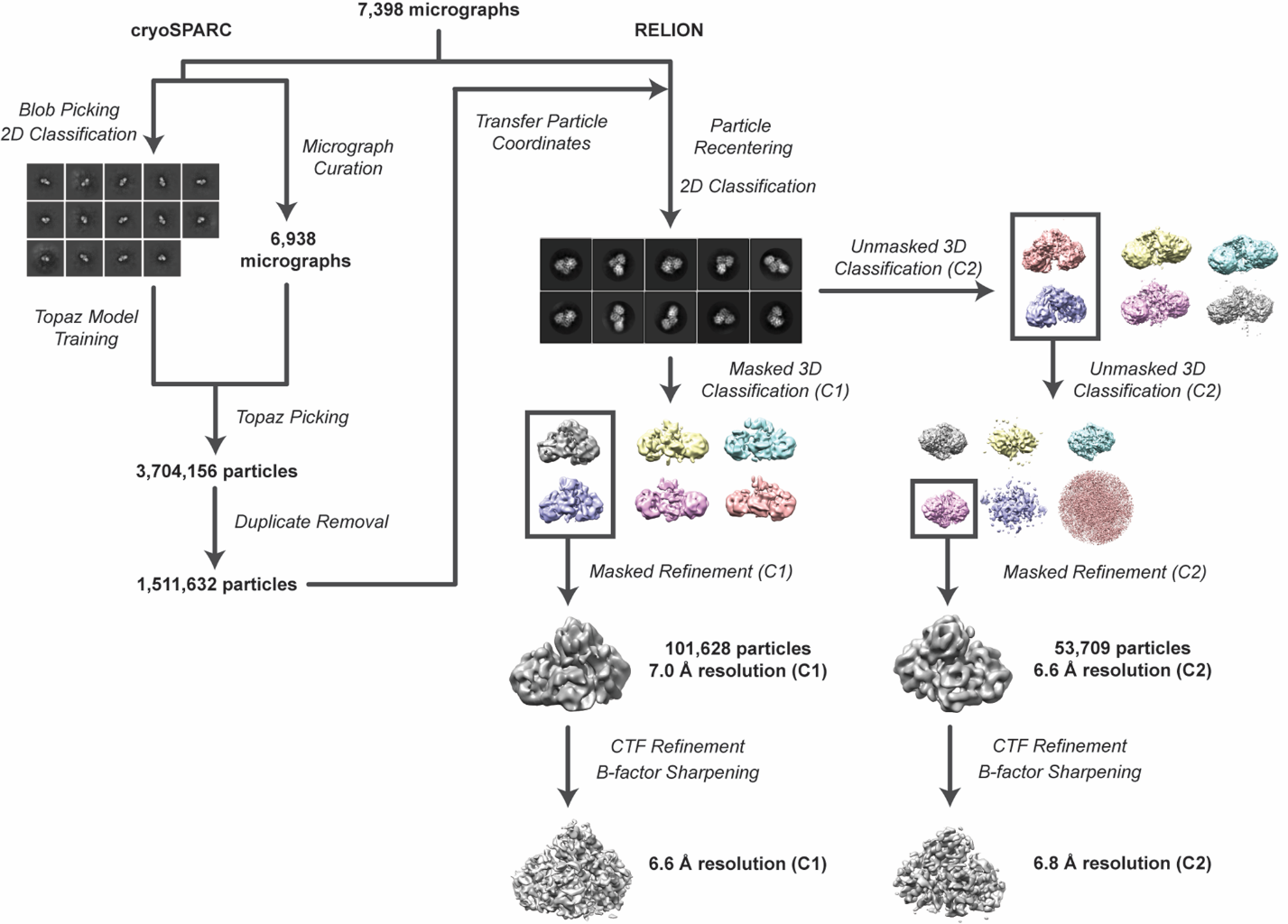


***Supplementary Figure S12.*** ***Simplified cryo-EM data processing workflow.***


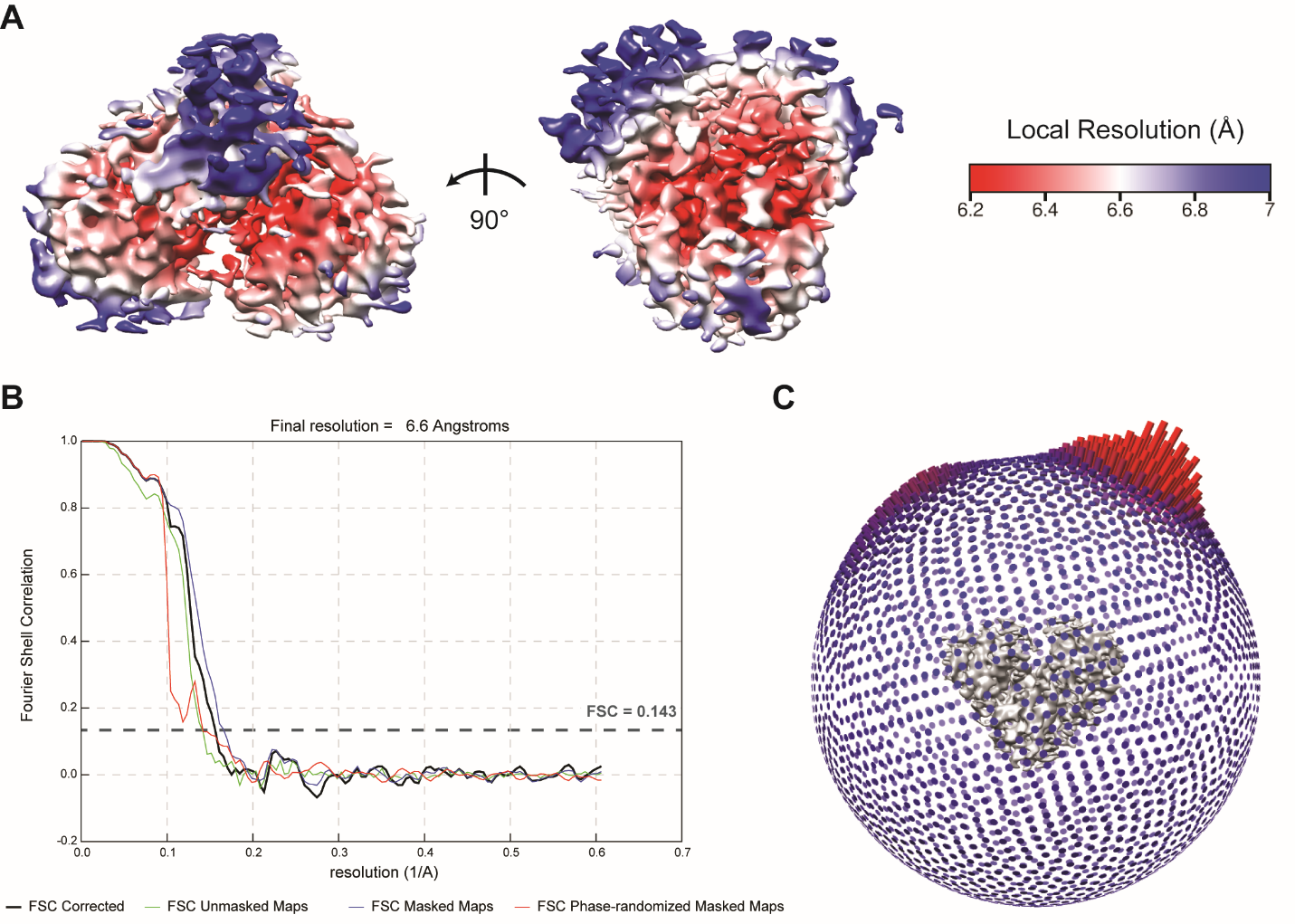


***Supplementary Figure S13.*** ***Cryo-EM map validation (C1 map).* (a)** Local resolution profile for the masked and sharpened C1 (no symmetry applied) holo*Tv*FDPF3 map. **(b)** Fourier shell correlation for the unmasked, masked, and phase-randomized masked C1 maps. The final corrected FSC is indicated in black. **(c)** Angular distribution of particle views in the final refined holo*Tv*FDPF3 C1 map.


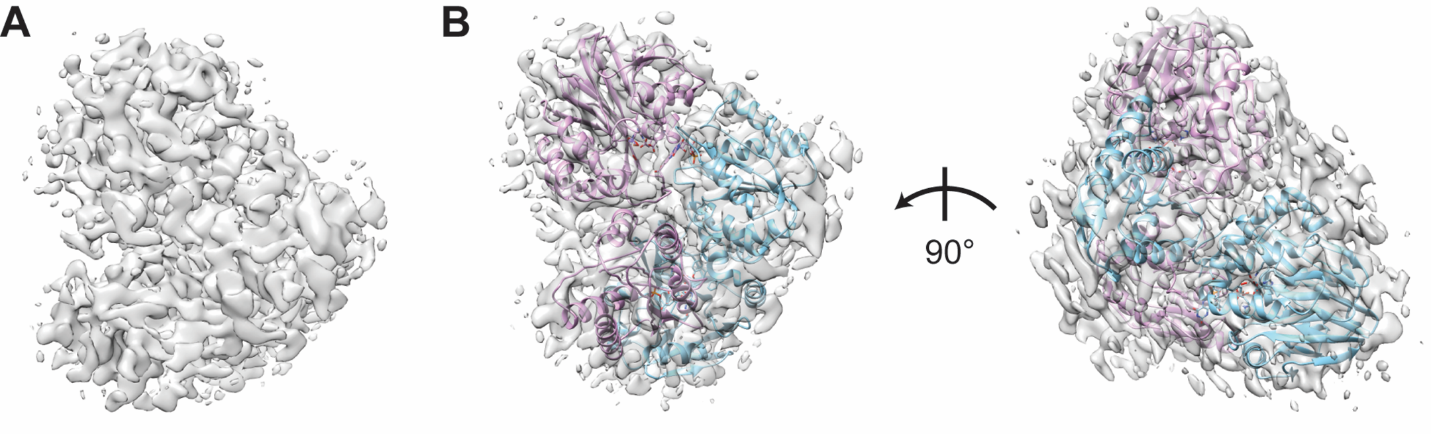


***Supplementary Figure S14. Homology model docking in C1 map.* (a)** C1-symmetric (no symmetry applied) electron density map for the holo*Tv*FDPF3 particles. **(b)** Homology models for the large and small subdomains of the holo*Tv*FDPF3 FDP-like domain docked into the C1-symmetric density map. The placement of the models indicates apparent C2 symmetry.


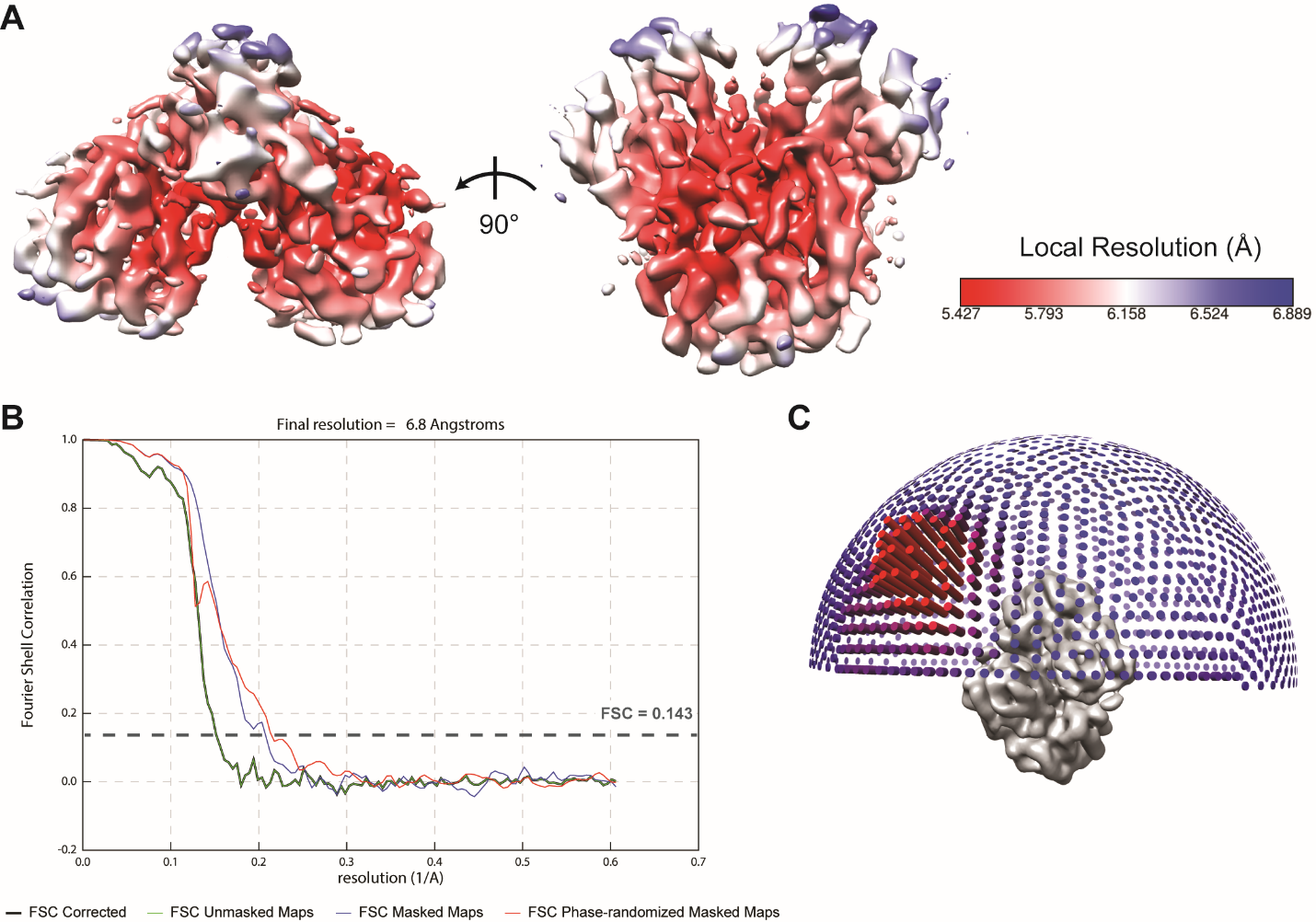


***Supplementary Figure S15.* *Cryo-EM Map Validation (C2 Map).* (a)** Local resolution profile for the masked and sharpened C2-symmetric holo*Tv*FDPF3 map. **(b)** Fourier shell correlation for the unmasked, masked, and phase-randomized masked C2 maps. The final corrected FSC is indicated in black. **(c)** Angular distribution of particle views in the final refined holo*Tv*FDPF3 C2 map.


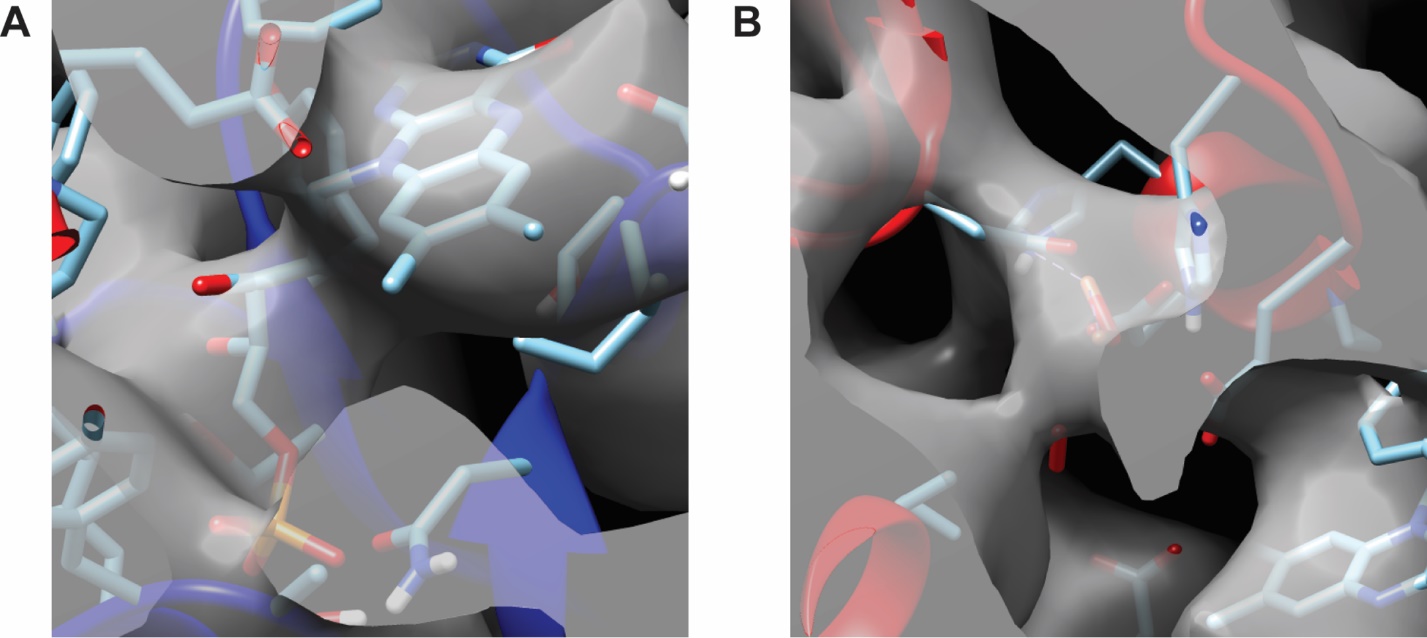


***Supplementary Figure S16. Ligand occupancy in electron density.*** Close-up view of the **(a)** FMN and **(b)** diiron ligands in the C2-symmetric electron density map.


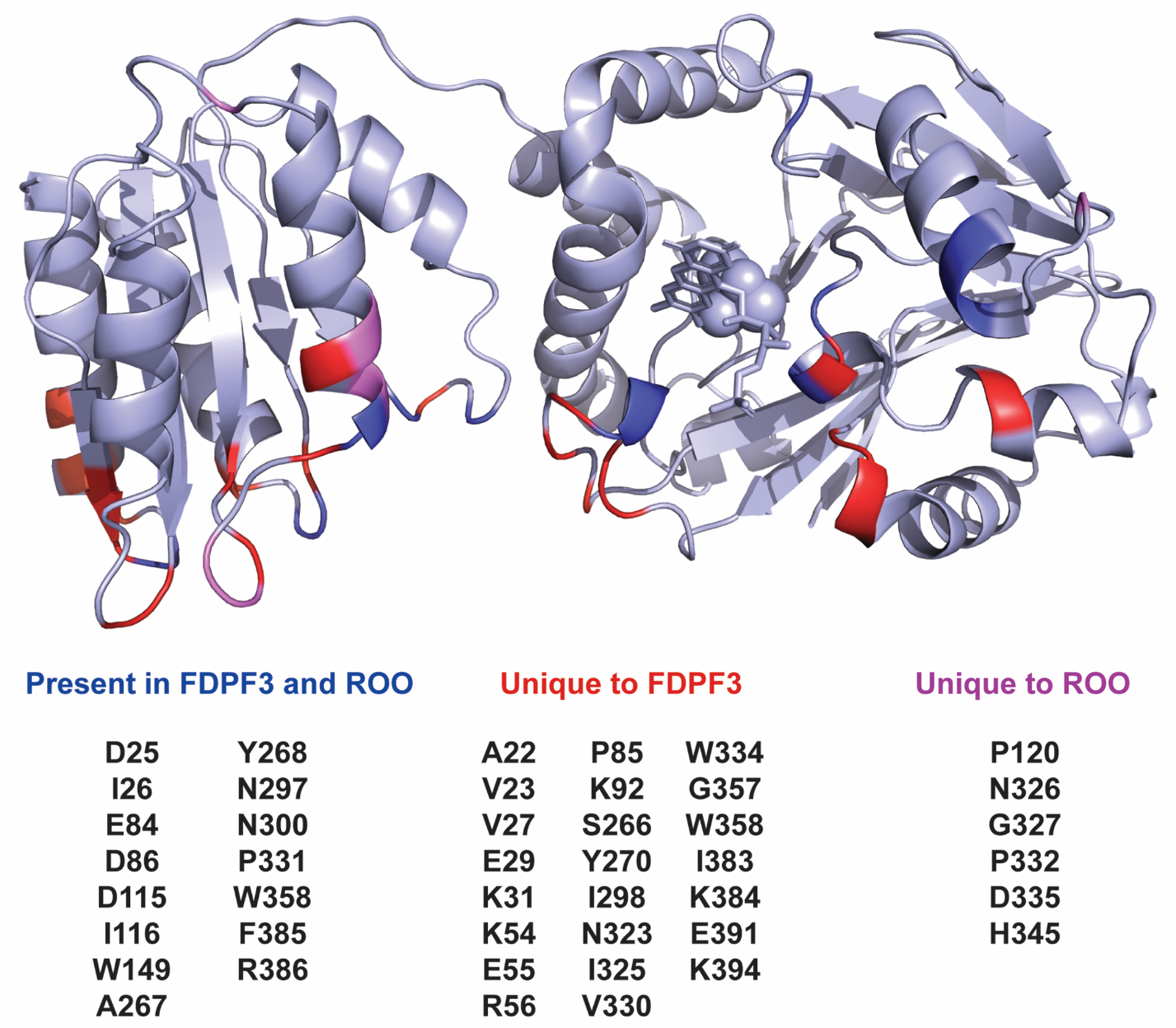


***Supplementary Figure S17.* *Inter-subunit contacts between holoTvFDPF3 protomers.*** Homology model of one subunit of the holo*Tv*FDPF3 FDP-like domain colored to identify interfacial residues (defined as residues in one subunit within 3.5 Å of any residue in the neighboring subunit). Residues colored blue form inter-protomer contacts in both the holo*Tv*FDPF3 model and the published structure of *D. gigas* ROO. Residues colored red form inter-protomer contacts unique to holo*Tv*FDPF3. Residues colored in purple form inter-protomer contacts in *D. gigas* ROO, but not in the model of holo*Tv*FDPF3. Residues are listed as numbered in holo*Tv*FDPF3.
