# Supplementary figures and images for: A natural fusion of flavodiiron, rubredoxin, and rubredoxin oxidoreductase domains is a self-sufficient water-forming oxidase of Trichomonas vaginalis
Source: J Biol Chem. 2022 Jun 30;298(8):102210. doi: 10.1016/j.jbc.2022.102210 (PMC9364112; doi:10.1016/j.jbc.2022.102210)

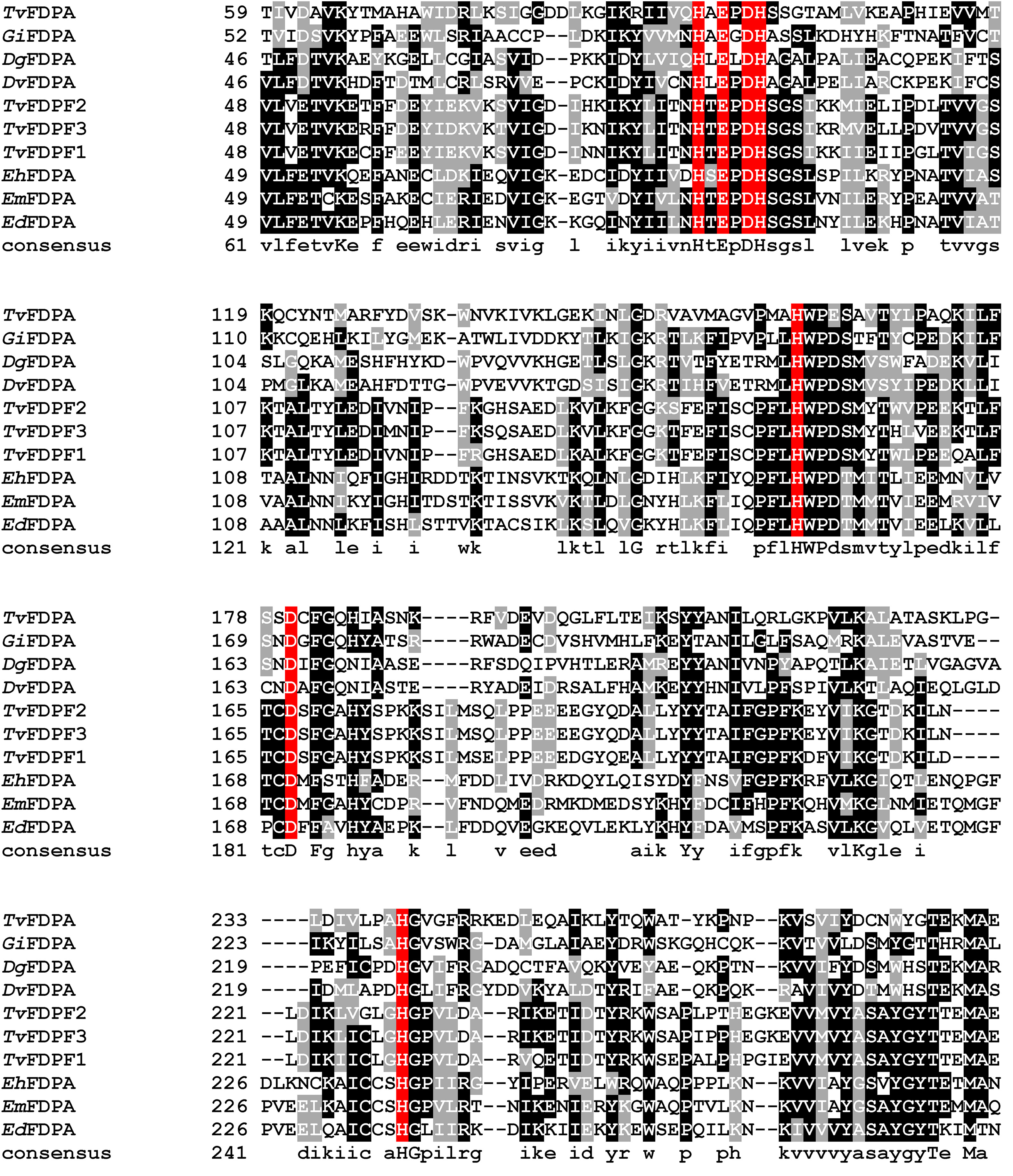

Supplement: Supplementary S1 [file figs1.jpg]

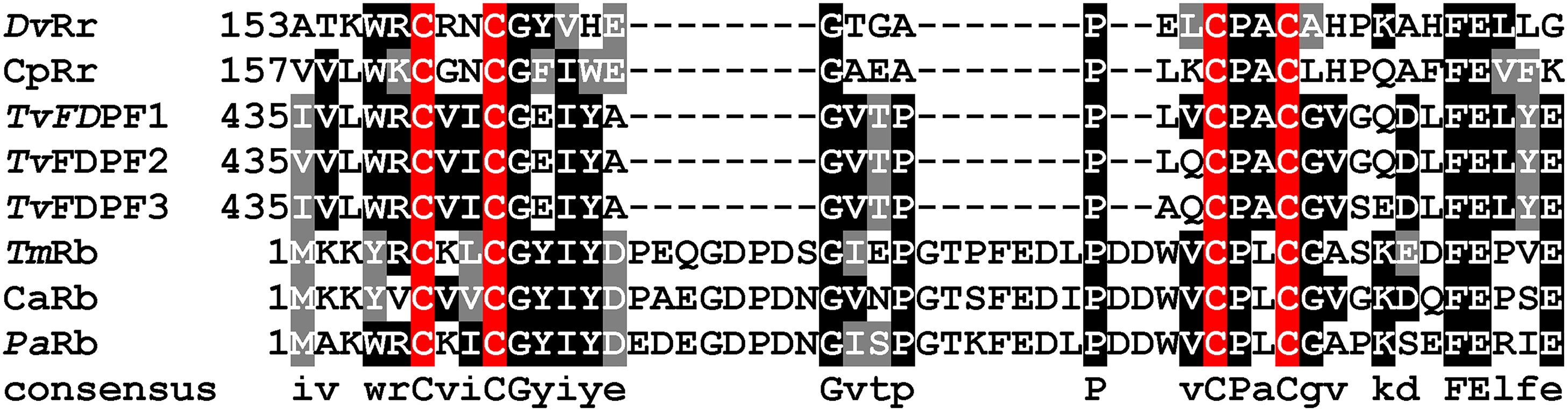

Supplement: Supplementary S2 [file figs2.jpg]

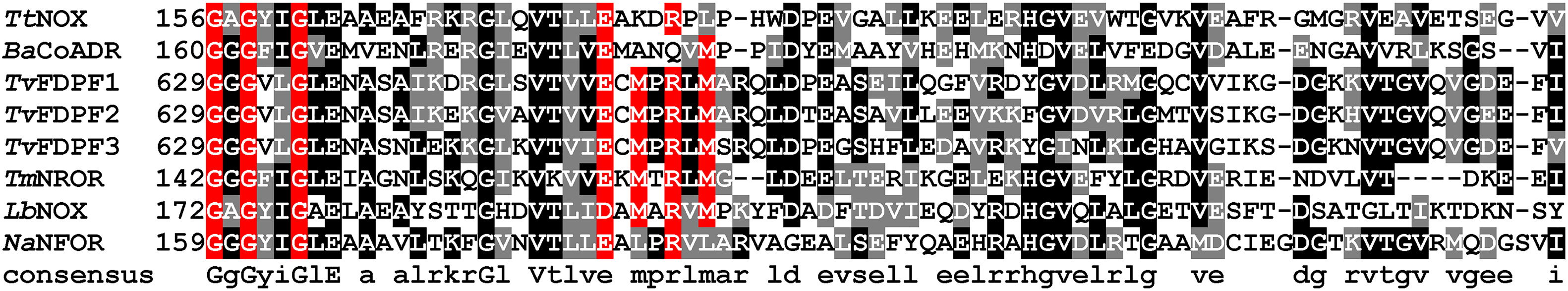

Supplement: Supplementary S3 [file figs3.jpg]

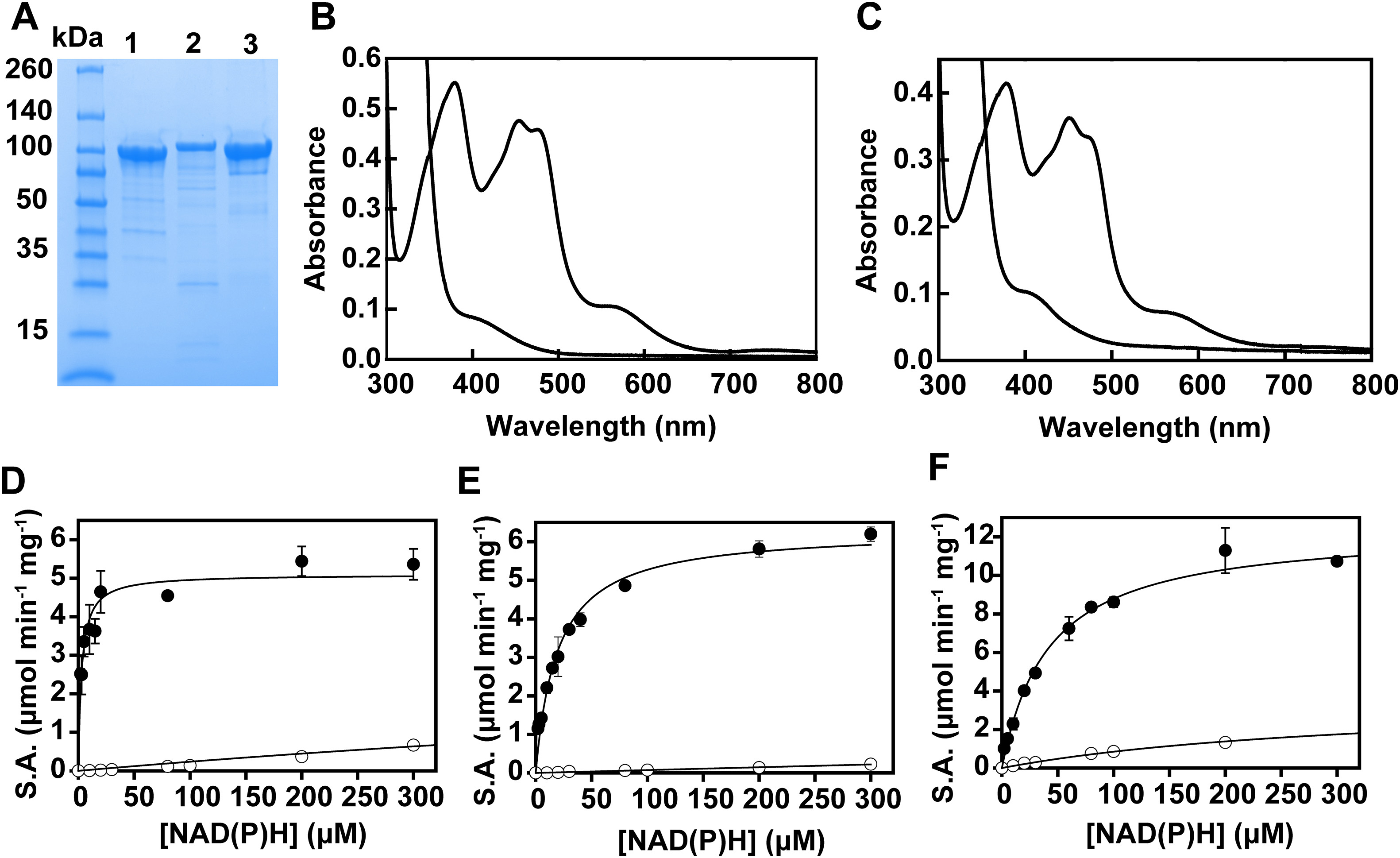

Supplement: Supplementary S4 [file figs4.jpg]

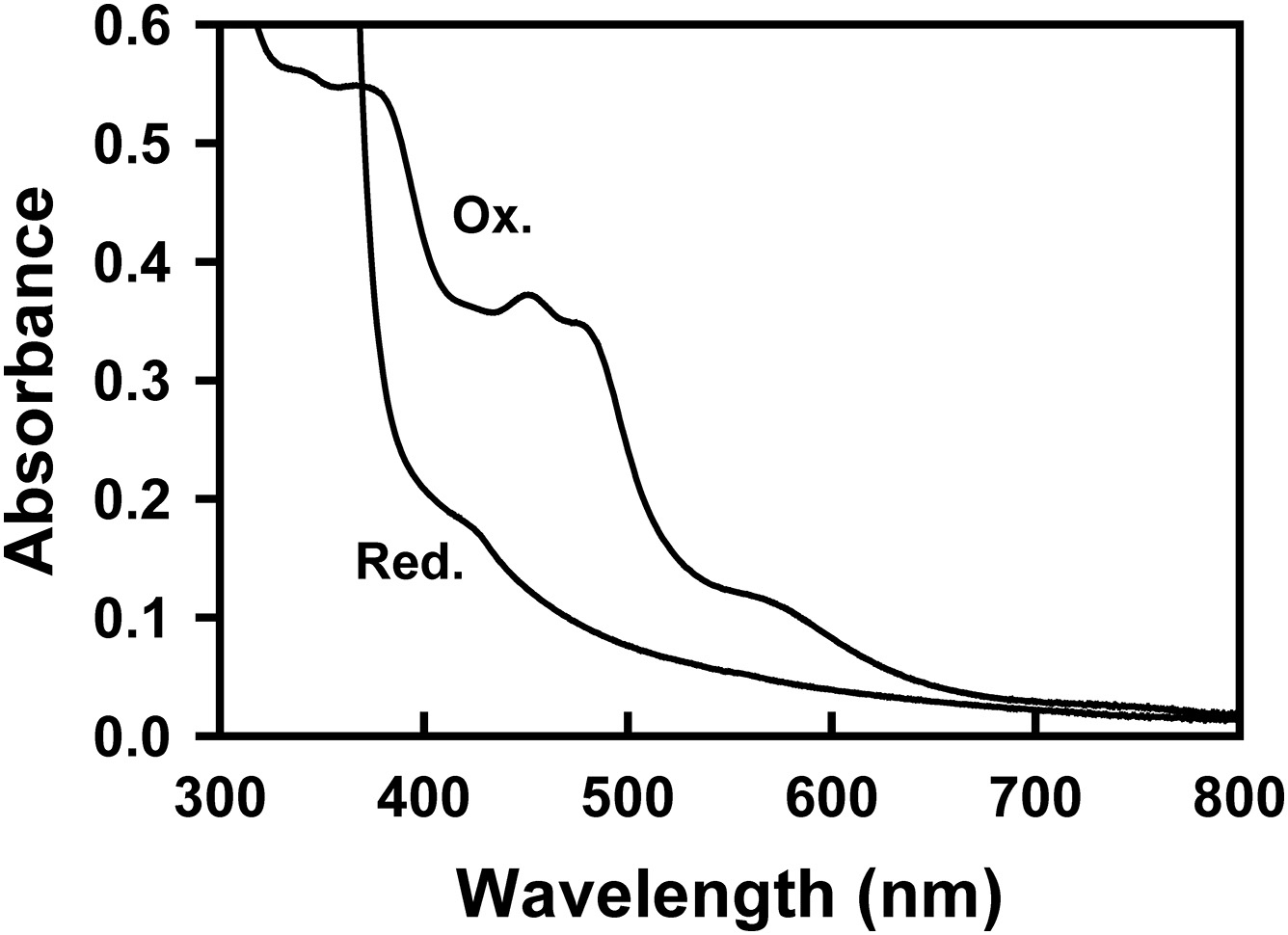

Supplement: Supplementary S5 [file figs5.jpg]

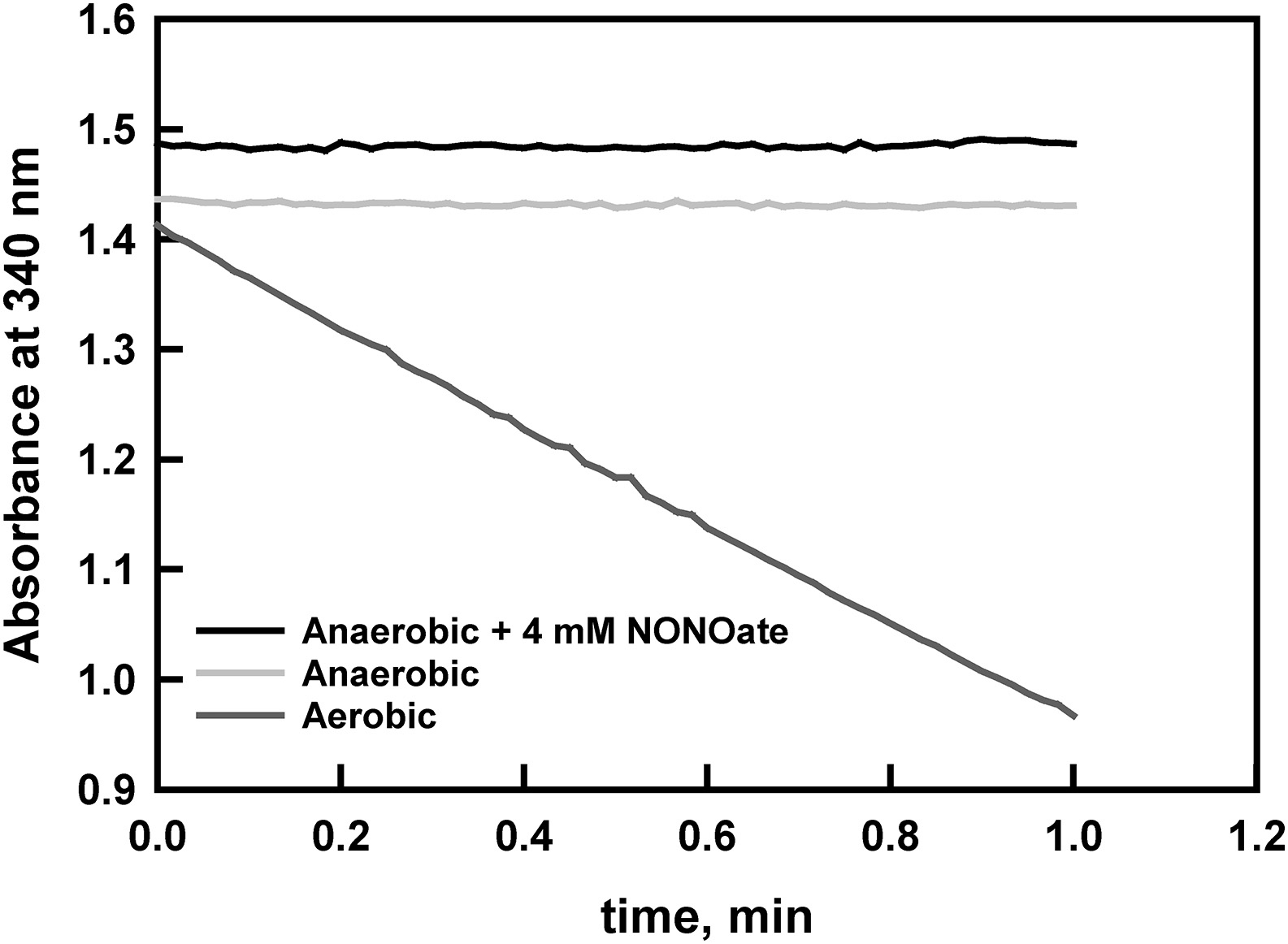

Supplement: Supplementary S6 [file figs6.jpg]

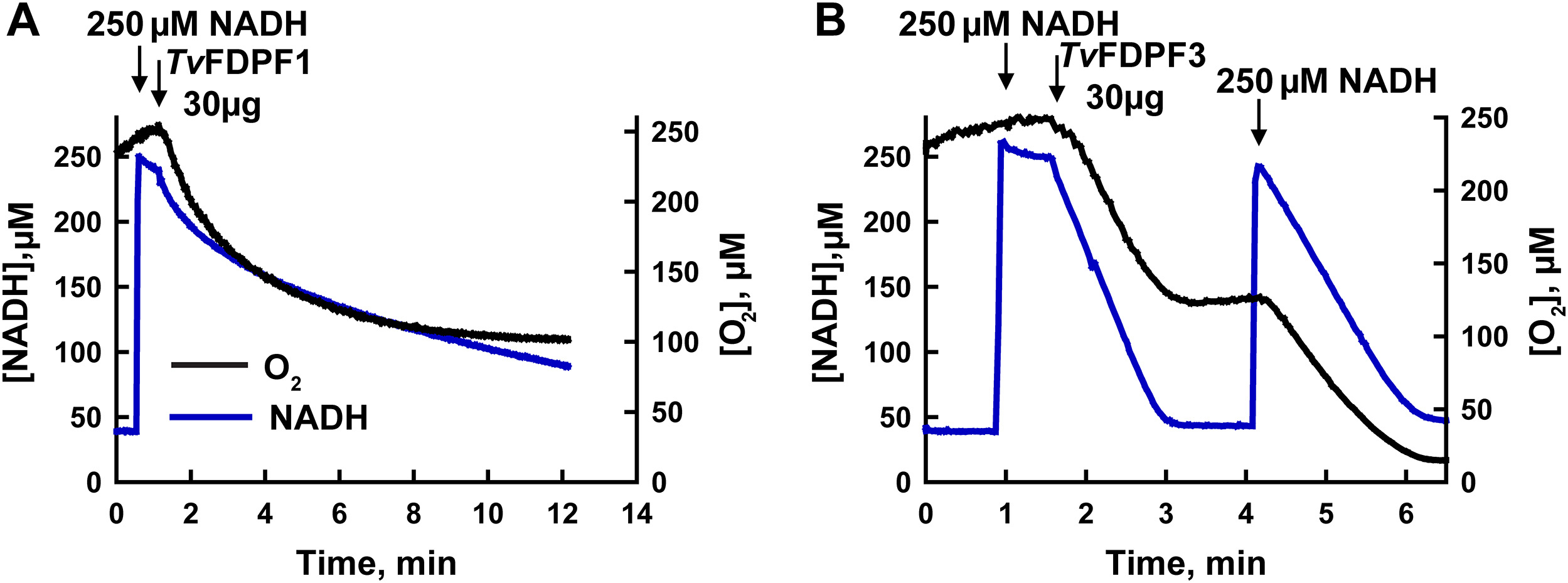

Supplement: Supplementary S7 [file figs7.jpg]

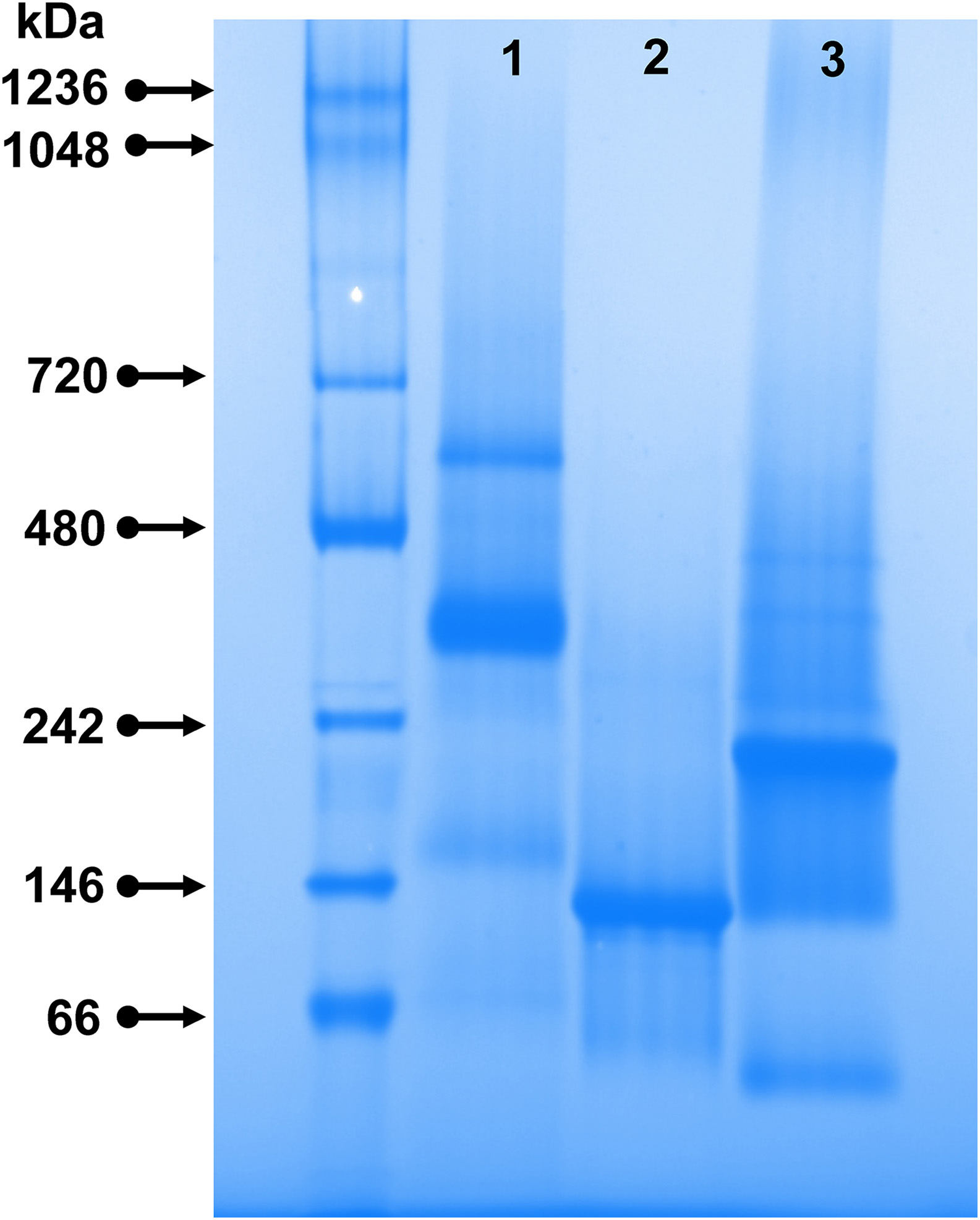

Supplement: Supplementary S8 [file figs8.jpg]

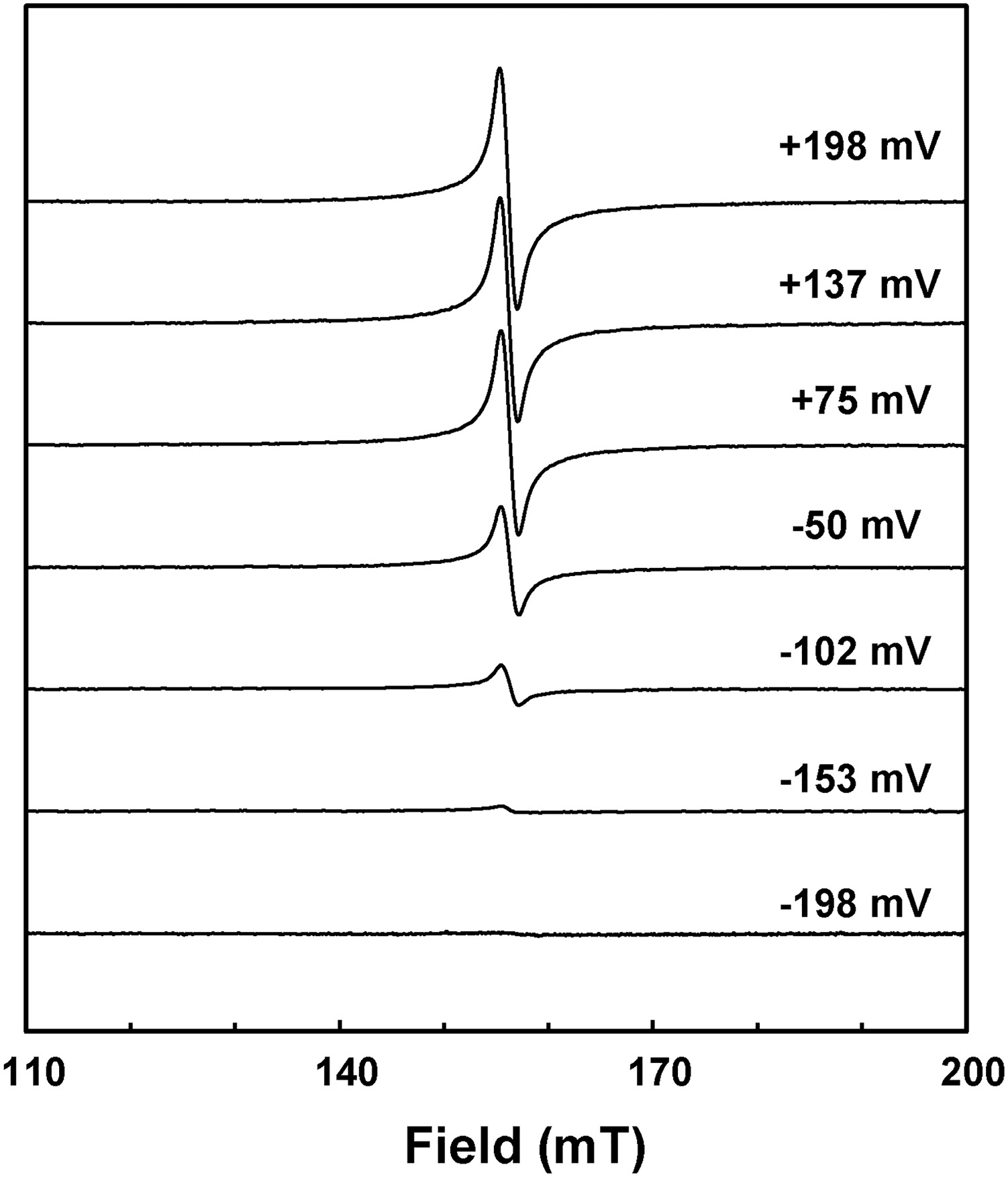

Supplement: Supplementary S9 [file figs9.jpg]

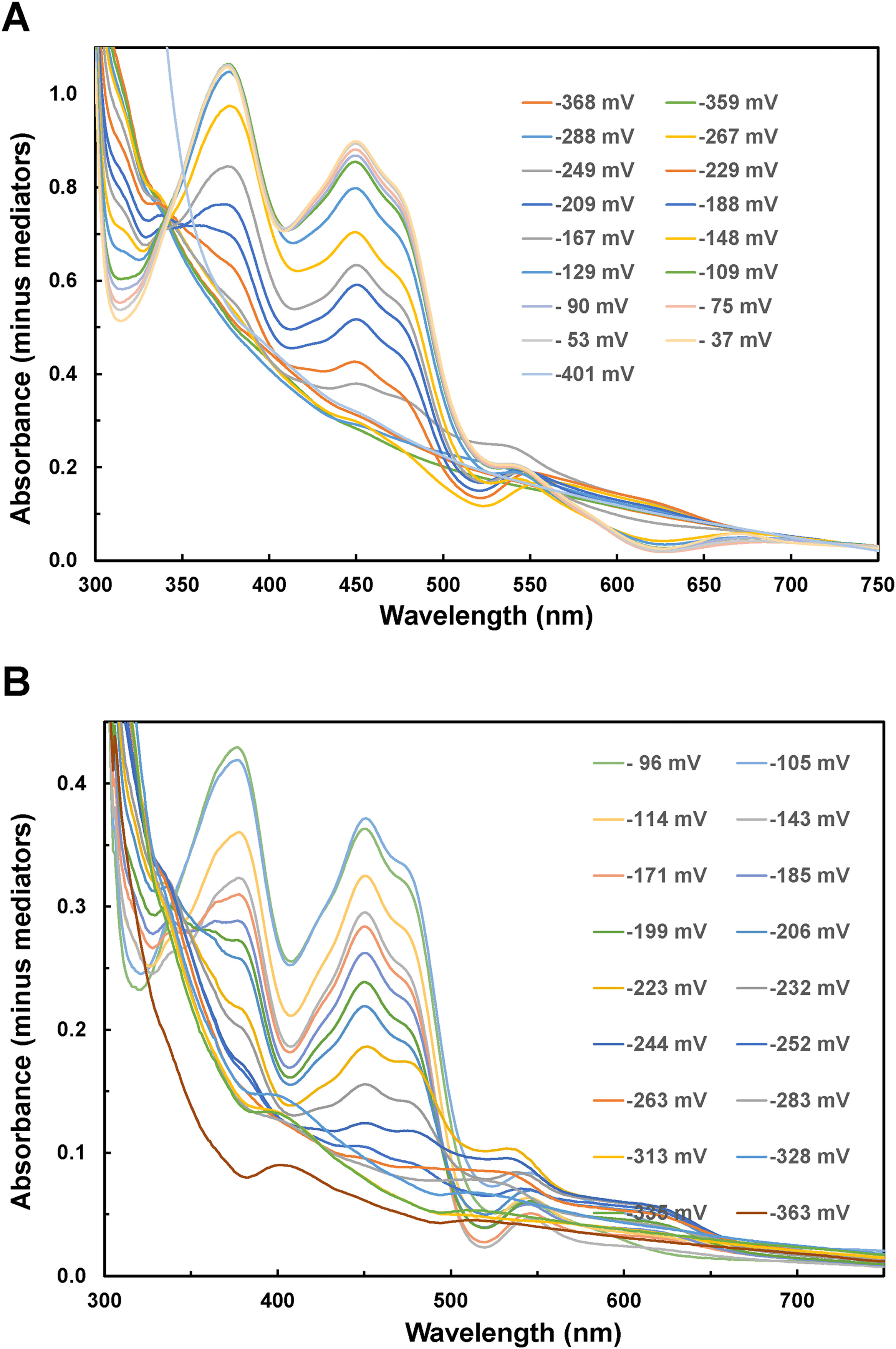

Supplement: Supplementary S10 [file figs10.jpg]

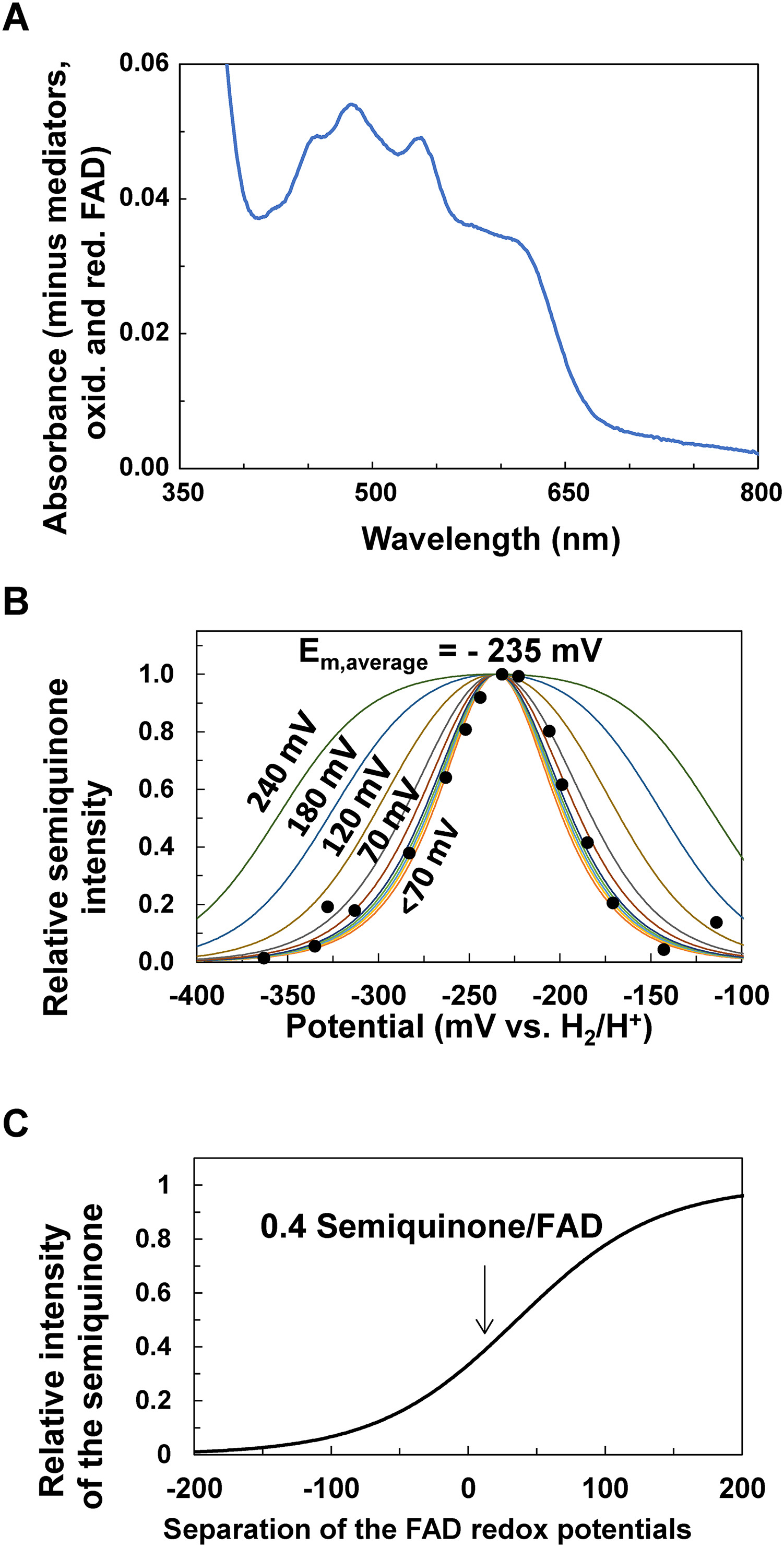

Supplement: Supplementary S11 [file figs11.jpg]

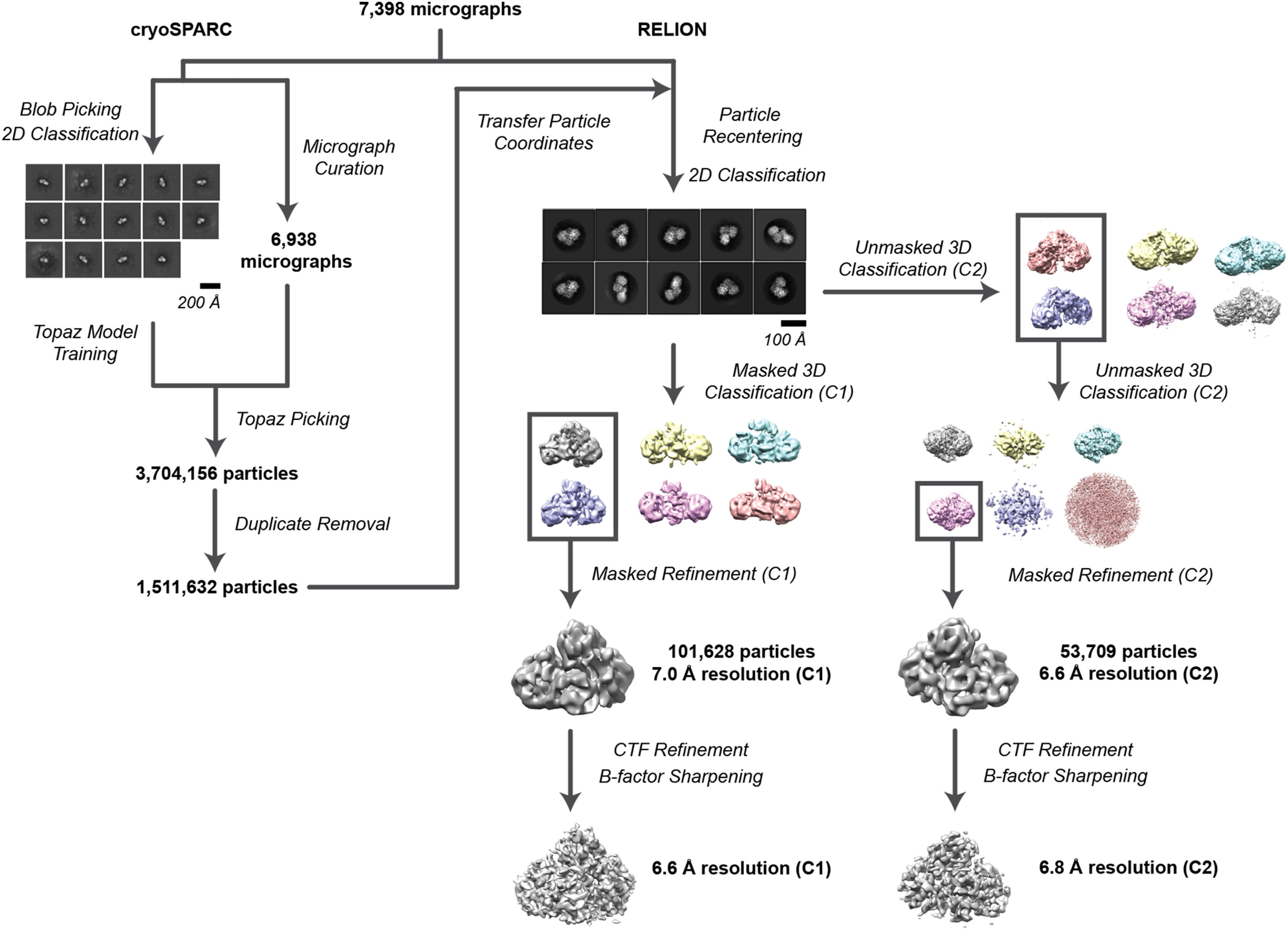

Supplement: Supplementary S12 [file figs12.jpg]

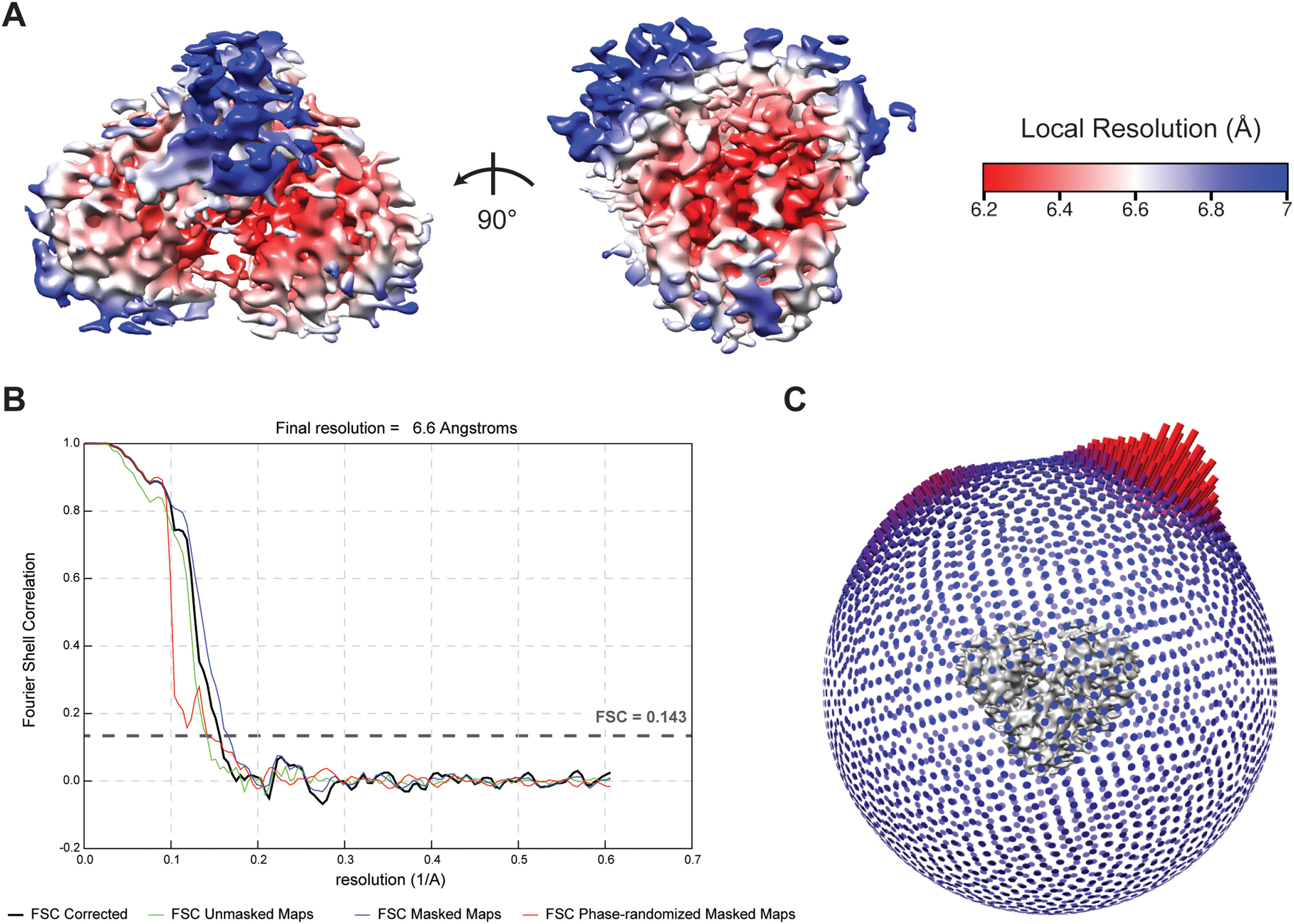

Supplement: Figure S13 [file figs13.jpg]

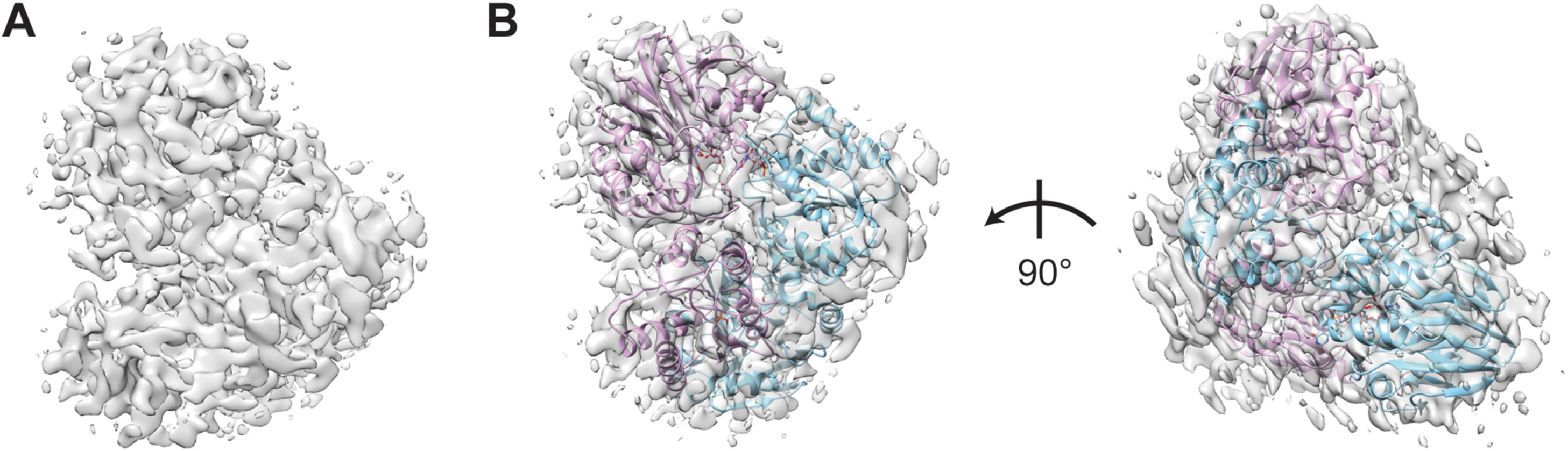

Supplement: Figure S14 [file figs14.jpg]

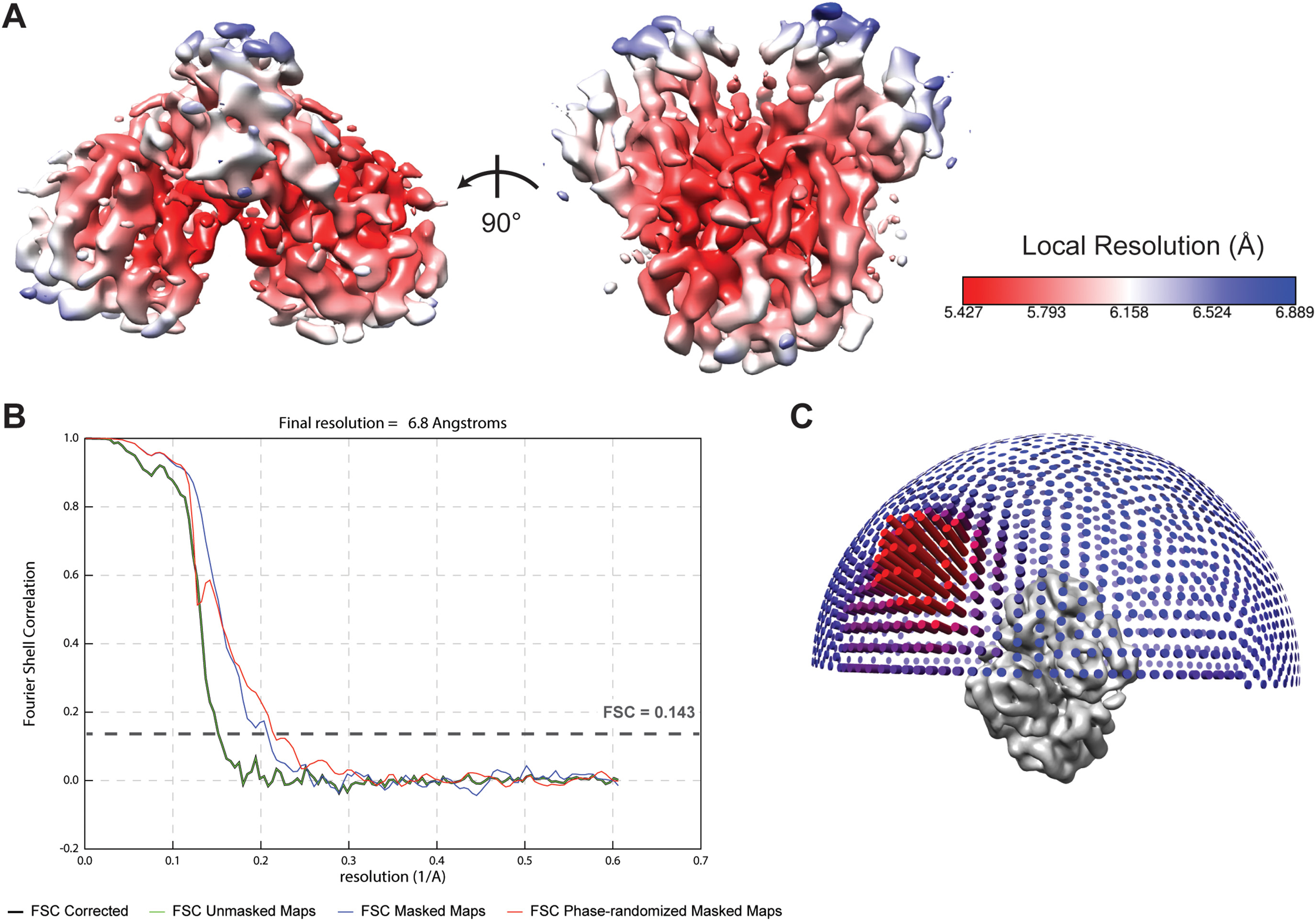

Supplement: Figure S15 [file figs15.jpg]

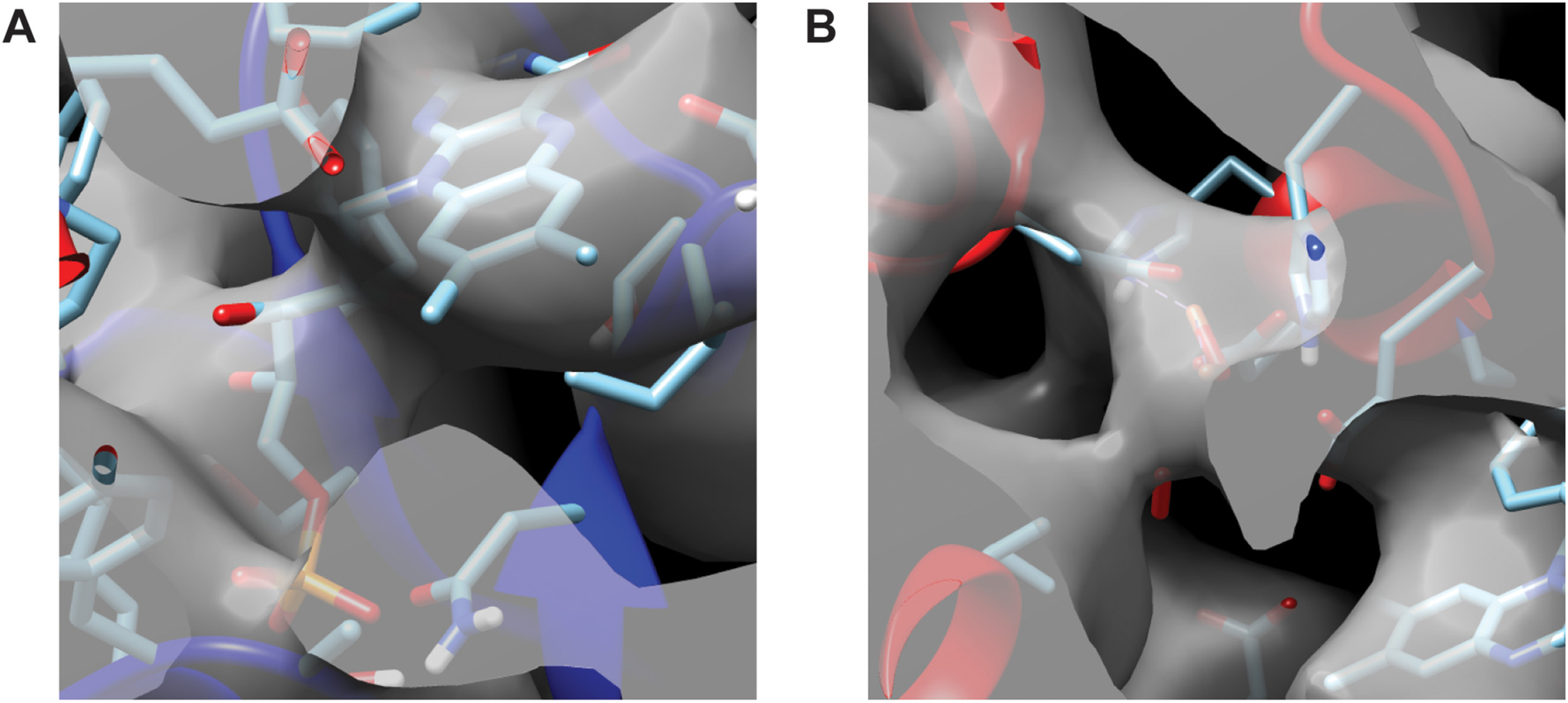

Supplement: Figure S16 [file figs16.jpg]

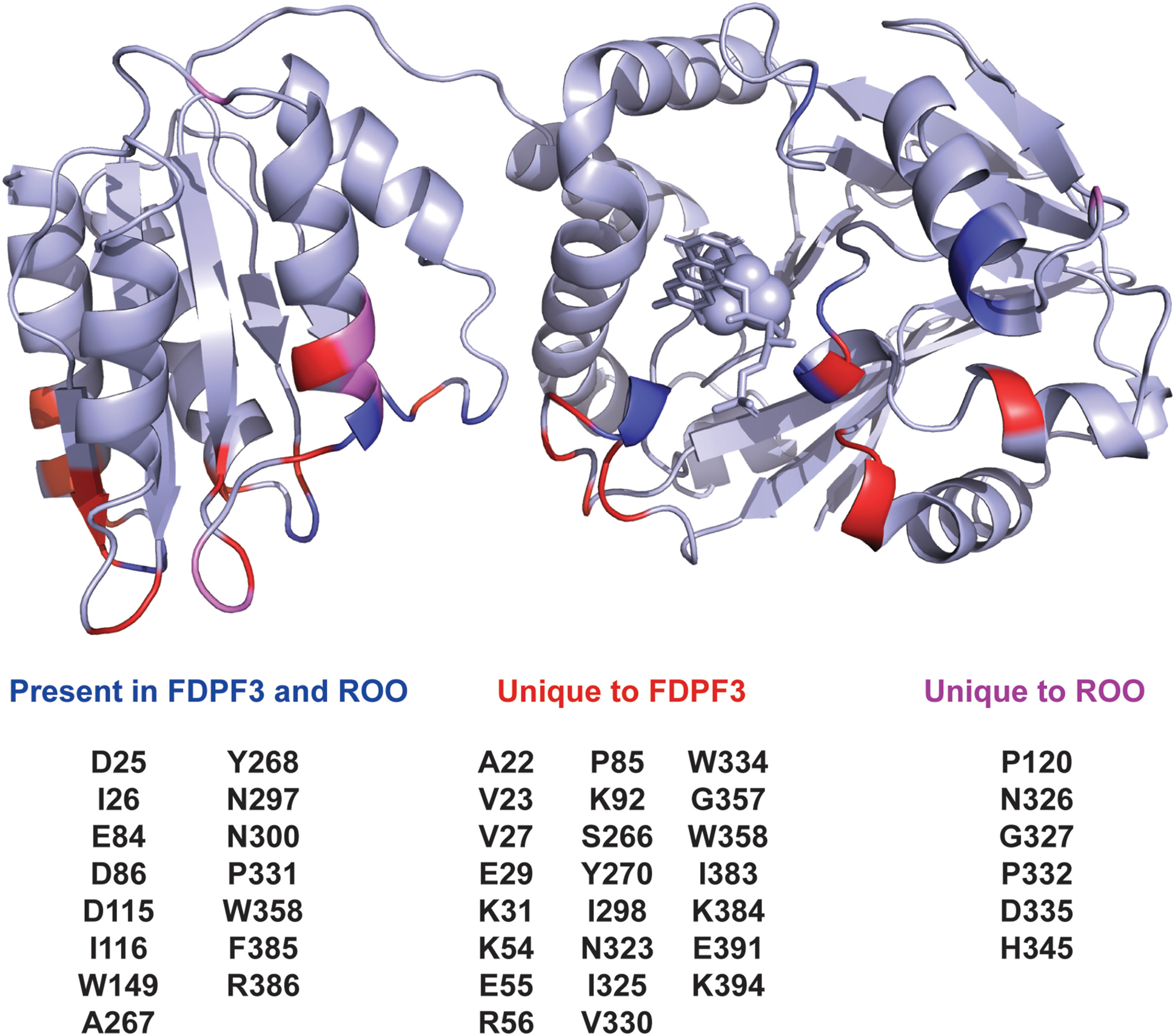

Supplement: Figure S17 [file figs17.jpg]
